# Epithelium-derived SCUBE3 promotes polarized odontoblastic differentiation of dental mesenchymal stem cells and pulp regeneration

**DOI:** 10.1186/s13287-023-03353-0

**Published:** 2023-05-15

**Authors:** Zijie Wang, Chuying Chen, Jiayi Zhang, Jiangdie He, Lin Zhang, Jiayuan Wu, Zhihui Tian

**Affiliations:** 1 Department of Stomatology, Nanfang Hospital, Southern Medical University, No. 1838, Guangzhou Road North, Baiyun District, Guangzhou, 510000 Guangdong China; 2 School of Stomatology, Southern Medical University, Guangzhou, Guangdong China; 3 Hospital of Stomatology, Zunyi Medical University, No. 143 Dalian Road, Huichuan District, Zunyi, 563000 China; 4 Special Key Laboratory of Oral Disease Research of Higher Education Institution of Guizhou Province, Zunyi, China; 5 Department of Histology and Embryology, School of Basic Medical Sciences, Southern Medical University, No. 1838, Guangzhou Road North, Baiyun District, Guangzhou, 510000 Guangdong China

**Keywords:** SCUBE3, Pulp–dentin regeneration, Odontoblastic differentiation, BMP2/Smad signalling, TGF/Smad signalling, Paracrine/autocrine

## Abstract

**Background:**

Signal peptide-CUB-EGF domain-containing protein 3 (SCUBE3), a secreted multifunctional glycoprotein whose transcript expression is restricted to the tooth germ epithelium during the development of embryonic mouse teeth, has been demonstrated to play a crucial role in the regulation of tooth development. Based on this, we hypothesized that epithelium-derived SCUBE3 contributes to bio-function in dental mesenchymal cells (Mes) via epithelium–mesenchyme interactions.

**Methods:**

Immunohistochemical staining and a co-culture system were used to reveal the temporospatial expression of the SCUBE3 protein during mouse tooth germ development. In addition, human dental pulp stem cells (hDPSCs) were used as a Mes model to study the proliferation, migration, odontoblastic differentiation capacity, and mechanism of rhSCUBE3. Novel pulp–dentin-like organoid models were constructed to further confirm the odontoblast induction function of SCUBE3. Finally, semi-orthotopic animal experiments were performed to explore the clinical application of rhSCUBE3. Data were analysed using one-way analysis of variance and t-tests.

**Results:**

The epithelium-derived SCUBE3 translocated to the mesenchyme via a paracrine pathway during mouse embryonic development, and the differentiating odontoblasts in postnatal tooth germ subsequently secreted the SCUBE3 protein via an autocrine mechanism. In hDPSCs, exogenous SCUBE3 promoted cell proliferation and migration via TGF-β signalling and accelerated odontoblastic differentiation via BMP2 signalling. In the semi-orthotopic animal experiments, we found that SCUBE3 pre-treatment-induced polarized odontoblast-like cells attached to the dental walls and had better angiogenesis performance.

**Conclusion:**

SCUBE3 protein expression is transferred from the epithelium to mesenchyme during embryonic development. The function of epithelium-derived SCUBE3 in Mes, including proliferation, migration, and polarized odontoblastic differentiation, and their mechanisms are elaborated for the first time. These findings shed light on exogenous SCUBE3 application in clinic dental pulp regeneration.

**Supplementary Information:**

The online version contains supplementary material available at 10.1186/s13287-023-03353-0.

## Background

Stem cell-based pulp–dentin regeneration may replace routine root canal treatment and has recently emerged as a promising therapeutic strategy [1, 2]. Human dental pulp stem cells (hDPSCs) are an easily accessible population with multipotency and self-renewal ability, properties that have led to their recognition as ideal seed cells for pulp–dentin regeneration [3]. However, previous studies have suggested that implanting decellularized scaffolds loaded only with hDPSCs is not efficient for real dentin–pulp complex regeneration [4, 5]. The odontoblastic differentiation potential of dental stem cells is vital for pulp regeneration and a prerequisite for dentin–pulp complex formation [6, 7]. Thus, it is imperative to optimize stem cell delivery strategies to enhance the odontoblastic differentiation of hDPSCs.

Many cytokines crucial for tooth development are expressed in a specific temporospatial pattern via epithelial–mesenchymal interactions [8]. Therefore, a further understanding of tooth development could contribute to strategies for pulp–dentin regeneration [9, 10]. Signal peptide-CUB-EGF domain-containing protein 3 (SCUBE3), a secreted multifunctional cell plasma membrane-anchored glycoprotein, is a member of the SCUBE family, sharing a distinct domain organization of at least five recognizable motifs. The specific CUB-like domain encoded by the *SCUBE3* gene can directly bind to the TGFβ/BMP receptor on the cell membrane as a ligand and cause corresponding biological effects [11]. A recent research reported that SCUBE3, which was expressed only in dermal papillae of growing, but not in resting follicles, was a new mesenchymal niche factor that activates hair growth via the interactions between epithelium and mesenchyme [12]. Previous studies have reported that *Scube1* and *Scube3* have dynamic reciprocal expressional patterns within the mesenchyme and epithelium during murine odontogenesis [13]. In brief, *SCUBE3* transcripts are only detected in the epithelial tissue and follow a specific and dynamic transcriptional pattern at the early stage of tooth formation [13, 14].

In a recent report, 18 individuals with biallelic inactivating variants in *SCUBE3* showed a consistent phenotype characterized by abnormal skeletal features, distinctive craniofacial appearance, and dental anomalies. Based on this finding, a novel disease caused by the defective function of *SCUBE3* and linked to processes that control abnormal tooth development was defined [15]. Besides, other studies have reported that the specific knockout of *SCUBE3* leads to a decrease in height, increase in width of ameloblasts, and loss of representative Tomes processes [16] and that changes in ameloblast morphology are related to enamel hypoplasia [17]. Considering the induction of ameloblasts is an indispensable factor for the differentiation of odontoblasts [18] and SCUBE3 is a secreted glycoprotein [11], we hypothesized that epithelium-derived SCUBE3 is involved in inducing odontoblastic differentiation of mesenchymal cells (Mes) during tooth development.

To the best of our knowledge, this study is the first to elaborate on the temporospatial expression of SCUBE3 in mouse embryonic and postnatal tooth germs and the function of epithelium-derived SCUBE3 in dental Mes. Our results demonstrate that epithelium-derived SCUBE3 translocates to Mes via a paracrine pathway, accelerates proliferation, migration, and polarized odontoblastic differentiation of Mes, and promotes vascularized pulp regeneration.

## Methods

### Cell isolation and culture

This study was approved by the institutional review board of the Stomatological Hospital, Southern Medical University (201806). Healthy premolars and third molars were collected from patients aged 18–22 years for orthodontic reasons. Written informed consent was obtained from all participants. After extraction, the teeth were immediately placed in culture medium and kept at a low temperature for delivery to the laboratory for further processing. hDPSCs were isolated as previously reported [19]. Briefly, pulp tissue was extracted from the teeth, minced, and digested with collagenase type I (3 mg/mL; Invitrogen, Paisley, UK) and dispase (4 mg/mL; Invitrogen) for 1 h at 37 °C. After filtration with 70-mm strainers and centrifugation at 1000 rpm for 5 min, cells were resuspended and cultured in low-glucose Dulbecco’s modified Eagle’s medium (Gibco-BRL, Gaithersburg, MD, USA) supplemented with 10% foetal bovine serum (FBS; Gibco-BRL), 100 U/mL penicillin, and 100 mg/mL streptomycin (Invitrogen). The medium was refreshed every 2–3 days. Cells were passaged when they reached 70–80% confluency, and all experiments were performed at passages 3–5.

Tooth germs of mandibular first molars from prenatal and new-born C57BL/6 mice were dissected surgically under a stereomicroscope, followed by isolation of mouse tooth germ epithelial cells (mEpi) and mouse tooth germ mesenchymal cells (mMes) following the method described in our previous study [20]. Briefly, the tooth germs were washed with phosphate-buffered saline (PBS), and the enzymatic reaction was conducted with dispase for 10.5 min at 37 °C. Subsequently, the reaction was stopped, and the tooth germ epithelial and mesenchymal tissues were separated using a 25-gauge needle under a microscope. After filtration and centrifugation, some of the tooth germ epithelial cells (Epi) and Mes were collected for RNA extraction, and the rest were resuspended and separately cultured in medium. When tooth germ Epi reached 70–80% confluency, the medium was replaced with dermal cell basal medium (ATCC; Manassas, VA, USA) for purification, while tooth germ Mes was cultured in normal medium.

The ameloblast-derived cell line LS-8 was purchased from the State Key Laboratory of Military Stomatology, Department of Dental Technical Laboratory, School of Stomatology, Fourth Military Medical University (Xi'an, China). LS-8 cells were initially cultured in normal medium to induce differentiation, and when tooth germ Epi reached 70–80% confluency, the medium was replaced with induction medium, which contained 50 mg/mL ascorbic acid and 10 mM β-glycerophosphate, and the cells were further cultured for 2 weeks.

### Co-culture system

Transwell permeable systems (Corning, New York, NY, USA) were used for the co-culture of Epi and Mes. LS-8 cells were seeded in the upper compartment, while mMes and hDPSCs were seeded separately in the lower compartment of the dish. After four days of co-culture, cells in the lower compartments were collected for subsequent experiments.

### Short hairpin (sh)RNA lentivirus infection

The shRNA-targeting *SCUBE3* lentivirus and shRNA negative control (NC) lentivirus were synthesized by Ribo Company (Guangzhou, China). For cell transfection, hDPSCs were seeded into six-well plates and transfected with lentiviral particles, including shSCUBE3#1–#3 and shNC. Untransfected cells were used as the blank control. After 72 h of transfection, the cells were collected to assess the efficiency of SCUBE3 silencing using quantitative reverse transcription polymerase chain reaction (RT-qPCR) and Western blotting. The shRNA sequences used in the present study are listed in Additional file 1: Table S1.

### Migration assays

After starvation, 2 × 10^5^ hDPSCs in medium with 1% FBS were plated in Transwell inserts (Corning), whereas the 10% FBS medium with rhSCUBE3 or SB431542 was added to the lower chambers. After 24 h of incubation, the migrated hDPSCs were fixed and stained using 0.5% crystal violet.

HDPSCs were seeded in a medium with rhSCUBE3 or SB431542. When they reached 90% confluency, hDPSCs were scratched using 200-μL pipette tips and photographed after 12 h.

### Proliferation assays

HDPSCs (1 × 10^3^) were plated into 96-well plates with rhSCUBE3 or SB431542 and measured using Cell Counting Kit-8 (CCK-8; Sigma-Aldrich, St Louis, MO, USA) at 450 nm absorbance on the indicated days. HDPSCs were treated with rhSCUBE3 or SB431542. When cells reached 60% confluency, a 5-ethynyl-2′-deoxyuridine (EdU) kit was applied per the manufacturer’s instructions.

### Alkaline phosphatase (ALP) staining

Normal medium was replaced with osteogenic inductive medium (OIM, Cyagen Biosciences, Guangzhou, China) in all groups when hDPSCs reached 70–80% confluency. After 2 weeks of osteogenic induction, the cells were fixed with 4% polyoxymethylene for 15 min, washed thrice with PBS for 5 min, and stained with BCIP/NBT ALP substrate (Beyotime Biotechnology, Guangzhou, China).

### Alizarin red (ARS) staining

ARS staining was conducted to analyse the mineralized nodules of the cells in different treatment groups. After 2 weeks of culturing in OIM, hDPSCs were fixed, washed, and stained with 0.5% ARS solution (Sigma-Aldrich) to visualize calcium deposition.

### Culture of dentin–pulp-like organoids

Dentin–pulp-like organoids were cultured as per a previous method [21]. According to the manufacturer’s instructions, hDPSCs at a density of 5 × 10^6^ cells/mL were mixed with Matrigel (Nitta Gelatin, Osaka, Japan) at a ratio of 1:1, placed onto a sheet of Parafilm (Bemis, Oshkosh, WI, USA), and incubated for 40 min until polymerization of matrices. The constructs were transferred and cultured in ultra-low adhesion petri dishes (Corning) for 10 days. The medium was then changed to OIM for different treatment groups. After 21 days of culturing, all organoids were harvested for further experiments.

### Immunofluorescent staining

For immunofluorescence staining, cultured cells and organoids were fixed for 20 min, permeabilized with 0.1% Triton X-100 for 15 min, washed thrice with PBS for 5 min, and blocked with 10% bovine serum albumin for 1 h. Cells were incubated with the primary antibody anti-SCUBE3 (ab189955; Abcam, Cambridge, UK), and the organoids were incubated with anti-DSPP (sc-73632; Santa Cruz Biotechnology, Santa Cruz, CA, USA) overnight. Sections of tooth root fragments were stained with DSPP (sc-73632; Santa Cruz) and α-tubulin (sc-8035; Santa Cruz). After washing thrice with PBS for 5 min, cells and organoids were, respectively, incubated with Dylight 594 (35560; Thermo Fisher Scientific, Waltham, UK) and CoraLite 488-conjugated (CL488-66122; ProteinTech Group, Chicago, IL, USA) secondary antibodies for 1 h in the dark. DAPI (Sigma-Aldrich) was used to stain the nuclei. Finally, the cells were observed under a Leica DMI4000 B fluorescence microscope (Leica Imaging Systems, Cambridge, UK). Organoids were transferred to observation dishes specified for confocal laser microscopy and examined with a confocal microscope (Carl Zeiss, Göttingen, Germany).

### RT-qPCR

Total cellular RNA was extracted from cells and tooth germ tissues using TRIzol reagent (Invitrogen), as per the manufacturer's instructions. Reverse transcription was performed with 1 mg of RNA using the PrimeScript RT reagent kit (Takara, Dalian, China). Quantitative PCR was conducted using the SYBR Premix Ex Taq II kit (Takara) on a CFX96 Touch Real-Time PCR Detection System (Bio-Rad, Hercules, CA, USA). The expression of the genes of interest was normalized to that of glyceraldehyde-3-phosphate dehydrogenase (*GAPDH*) in the same samples. The reactions were performed in triplicate, and three independent experiments were performed. The primer sequences used are listed in Additional file 1: Table S2.

### Western blot analysis

Total cell proteins were lysed using radioimmunoprecipitation assay (Beyotime) according to the manufacturer’s protocol. Proteins in the conditioned medium were extracted using a liquid sample total protein extraction kit (Solarbio, Beijing, China). Western blotting was performed as previously described [10]. The primary antibodies used were anti-SCUBE3 (ab189955; Abcam), anti-AMBN (orb155652; Biorbyt, Cambridge, UK), TGFβR1 (ab31013; Abcam), anti-DSPP (sc-73632; Santa Cruz), anti-DMP1 (ab103203; Abcam), anti-OPN (ab8448; Abcam), anti-OSX (ab13418; Abcam), anti-BMP2 (ab14933; Abcam), anti-BMPR1A (ab264043; Abcam), anti-*p*-SMAD1/5 (9516; Cell Signalling Technology, Boston, MA, USA), and anti-SMAD1 (D59D7; Cell Signalling Technology). Anti-GAPDH (Rayantibody, Beijing, China) was used as an internal control. Antibody binding was detected using an enhanced chemiluminescence kit (Millipore, Billerica, MA, USA). The intensity of the bands was quantified using the ImageJ software (NIH, USA).

### Preparation of root fragments of human teeth

Decellularized tooth root fragments were prepared according to a previous report [21]. In brief, the tooth root fragments were sectioned into 5-mm-thick slices using a high-speed dental handpiece. The canals were enlarged to 3 mm in diameter using ProTaper files (Dentsply-Maillefer, Ballaigues, Switzerland) to remove pulp tissue and partial dentin, soaked in 5% ethylene diamine tetraacetic acid (EDTA) for 5 min, and ultrasonicated for 10 min, followed by sealing one of the open endings with mineral trioxide aggregate (Dentsply Sirona, Yorktown, VA, USA). Tooth root fragments were stored in PBS containing 2% penicillin/streptomycin (Invitrogen) at 4 °C and disinfected using UV sterilization before use. According to the manufacturer's instructions, hDPSCs at a density of 5 × 10^5^ cells/mL were mixed with Matrigel (Nitta Gelatin) at a ratio of 1:1, injected into the canal cavity, and incubated at 37 °C for 30 min for the subsequent experiments.

### Subcutaneous implantation into nude mice

Surgical procedures were performed as formerly reported [10]. In short, in 6-week-old immune-compromised nude mice (n = 12 in each group) which were purchased from Southern Medical University Laboratory Animal Science and Technology Development Co., Ltd (Guangzhou, CN), general anaesthesia was induced and maintained by intraperitoneal injection of 1.2 μL of 1% pentobarbital sodium (Merck, Darmstadt, Germany). A single hydrogel-hDPSC-filled fragment was transplanted subcutaneously into the dorsal side of each mouse. All nude mice were euthanized by cervical dislocation under anaesthesia four weeks post-surgery, and all fragments were harvested. This manuscript adheres to the ARRIVE guidelines for the reporting of animal experiment which improves the reporting of research involving animals.

### Histological assessment

Tooth root fragments were fixed at 4 °C for 48 h and decalcified for 12 weeks using a 10% EDTA–2Na solution (pH 7.4) on an orbital shaker at room temperature. Embryonic day 12.5 (E12.5), E14.5, E16.5, E18.5, postnatal day 3 (P3), P7, and P14 mice were killed to collect mandible samples as reported previously [22]. Tooth root fragments and mandible samples were embedded in paraffin, sectioned at a thickness of 5 μm, and subjected to haematoxylin and eosin and Masson’s trichrome staining (Solarbio). Histomorphology was observed using the Scanscope CS and Image Scope software (Aperio, Sausalito, CA, USA). The ImageJ software was used for quantitative analysis.

### Immunohistochemical staining

Immunohistochemical analysis was conducted according to a standard protocol. Briefly, samples were incubated with anti-SCUBE3 (ab189955; Abcam) and anti-mitochondria (ab92824; Abcam) as primary antibodies, followed by washing and incubation with horseradish peroxidase-conjugated secondary antibodies. Histometric observations were performed as described for the histological assessment.

### Statistical analysis

Data were statistically analysed using SPSS software (version 20.0; IBM, Armonk, NY, USA) or GraphPad Prism 8 (GraphPad Software, Inc., La Jolla, CA, USA). Data are presented as mean ± standard deviation (SD) from at least three independent experiments. Differences among groups were compared using one-way analysis of variance (ANOVA) with t-tests. Statistical significance was set at *P* < 0.05.

## Results

### Epithelium-derived SCUBE3 translocates to dental mesenchymal cells via a paracrine pathway

**Fig. 1:**
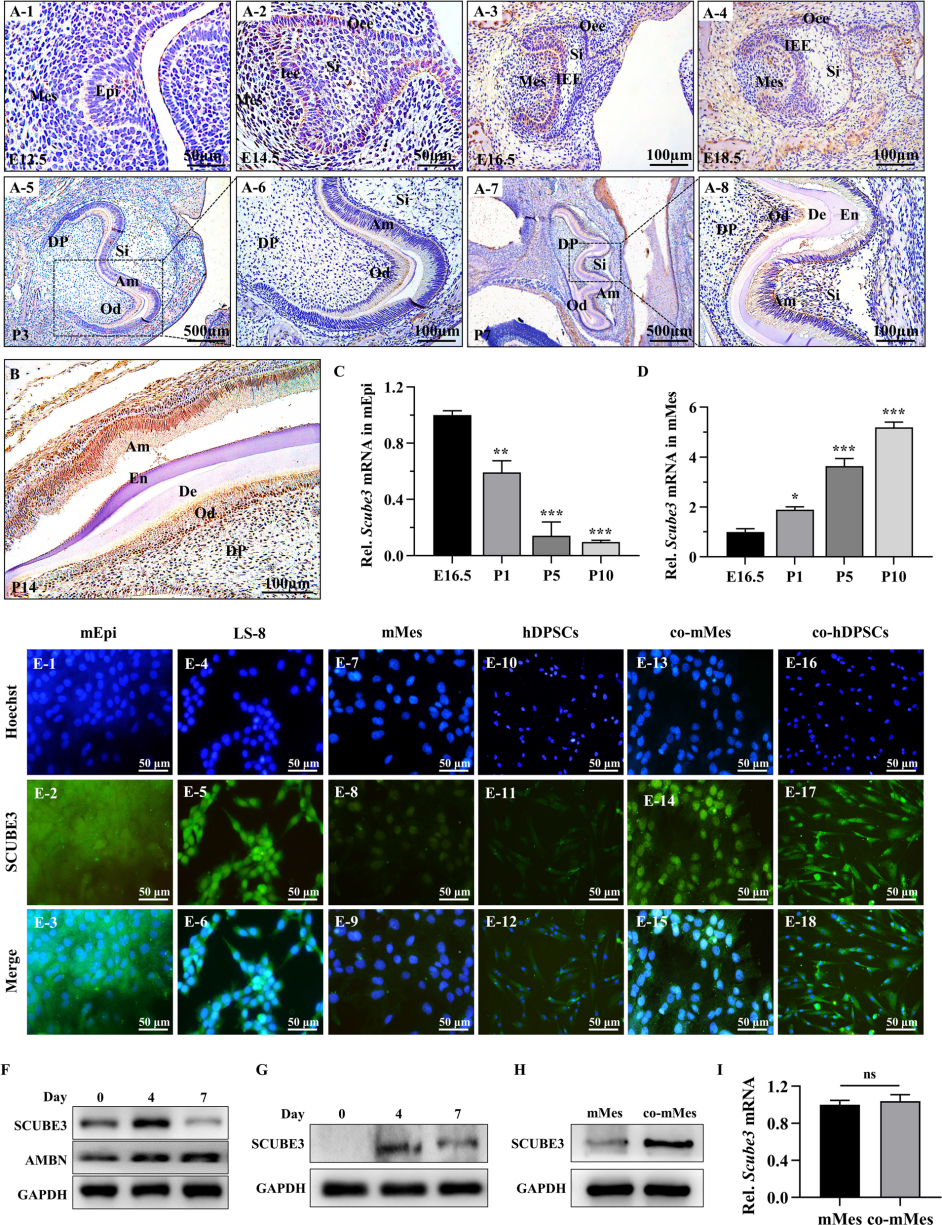
SCUBE3 is expressed during tooth development and translocated from Epi to Mes via the paracrine pathway. **A** SCUBE3 expression in mouse mandibular first molar tooth germ was detected using immunohistochemistry during tooth development on embryonic day 12.5 (E12.5), E14.5, E16.5, E18.5, and postnatal days 3 (P3) and P7. Boxed areas in (A6, 8) are shown at higher magnification than those in (A5, 7). **B** Immunohistochemistry for SCUBE3 in mouse incisors on P14. **C**, **D** *SCUBE3* transcript level in epithelial and mesenchymal tissues of the tooth germ on E16.5, P1, P5, and P10 was evaluated using RT-qPCR. **E** Immunofluorescence staining of dental Epi and Mes from mouse mandibular first molar tooth germ, LS-8 cells, co-cultured dental Mes, and co-cultured hDPSCs. **F** SCUBE3 expression in whole-cell lysates of LS-8 cells on the indicated days of ameloblastic differentiation was evaluated using Western blot analyses. Full-length blots/gels are presented in Additional file 3. **G** Western blot of secretory SCUBE3 in the conditioned medium of LS-8 during ameloblastic differentiation. Full-length blots/gels are presented in Additional file 3. **H** Expression of SCUBE3 protein in the untreated mMes and co-cultured mMes was evaluated using Western blot. Full-length blots/gels are presented in Additional file 3. **i** Expression of *SCUBE3* mRNA in the untreated mMes and co-cultured mMes was evaluated using RT-qPCR. *n* = 3 independent biological samples. *P < 0.05, **P < 0.01, ***P < 0.001. Am, ameloblasts. Dp, dental papilla. De, dentin. En, enamel. Epi, epithelial cells. Iee, inner enamel epithelium. Mes, mesenchymal cells. Si, stratum intermedium. Od, odontoblasts. Oee, outer enamel epithelium. hDPSC, human dental pulp stem cell. ECM, extracellular matrix

Here, we analysed the spatiotemporal expression pattern of SCUBE3 in the developing tooth germ using immunohistochemistry. SCUBE3 expression was initially present in the tooth germ epithelium at the bud stage (E12.5) and continuously increased thereafter (Fig. 1a1). During the cap stage at E14.5, SCUBE3 was strongly expressed in the whole epithelial tissue, including Epi and the extracellular matrix (ECM), whereas it was slightly expressed in the mesenchymal tissue, mainly in the ECM (Fig. 1a2). At the early bell stage (E16.5), SCUBE3 was gradually downregulated in the external enamel epithelium and stratum intermedium, while it was still strongly expressed in the internal enamel epithelium. Additionally, SCUBE3 was more highly expressed in mesenchymal tissue in the early bell than in the cap stage and was present in a small number of Mes (Fig. 1a3). During the late bell stage (E18.5), SCUBE3 expression in the epithelium was gradually attenuated, and SCUBE3 localized to the internal enamel epithelium, while its expression increased in the mesenchyme (Fig. 1a4). With further development until P3 (Fig. 1a5, 6) and P7 (Fig. 1a7, 8), a stronger expression was observed in mature odontoblasts, and the expression was significantly weaker in mature ameloblasts than in internal enamel cells at the early differentiation stage. Our results confirmed that SCUBE3 expression in the epithelium was reduced during the differentiation of internal enamel cells into ameloblasts, consistent with the presence of SCUBE3 transcripts in a previous report [13]. However, it was gradually upregulated in Mes, accompanied by odontoblastic differentiation. This temporospatial pattern of SCUBE3 expression was also observed in the cervical loop of the incisor and its vicinity (Fig. 1b).

Considering that SCUBE3 is a secreted protein, to investigate the origin of SCUBE3 in Mes, we quantified *Scube3* transcript expression in E16.5, P1, P5, and P10 tooth germ epithelial and mesenchymal tissues. *Scube3* was hardly expressed in the prenatal mesenchyme, suggesting that the SCUBE3 protein observed in the immunohistochemical sections of Mes was translocated from the epithelium. Remarkably, *Scube3* expression was strongly present but gradually reduced in the epithelial tissue of the postnatal tooth germ (Fig. 1c). This expression trend is consistent with the SCUBE3 protein level in the LS-8 (used as a model of tooth germ epithelial cells) ameloblast differentiation model (Fig. 1f). Secreted SCUBE3 was detected in the conditioned medium from day 0, which increased on day 4, and decreased thereafter (Fig. 1g), suggesting that mEpi produced and released SCUBE3 during ameloblastic differentiation. Remarkably, SCUBE3 was hardly expressed in the prenatal mMes but increased in the differentiating mMes of postnatal tooth germ (Fig. 1d). Immunofluorescence staining showed strong SCUBE3 expression in primary tooth germ Epi (Fig. 1e1–3) and LS-8 (Fig. 1e4–6), but weak expression in tooth germ Mes (Fig. 1e7–9) and hDPSCs (Fig. 1e10–12). Interestingly, after their co-culture, clear SCUBE3 expression was observed in tooth germ Mes (Fig. 1e13–15) and hDPSCs (Fig. 1e16–18). Western blotting also showed significantly higher SCUBE3 expression in co-cultured mMes than mMes cultured alone (Fig. 1h). However, there is no significant difference in SCUBE3 transcript levels between them (Fig. 1i), demonstrating the increased SCUBE3 protein in co-cultured mMes was translocated from mEpi via paracrine signalling.

### Exogenous SCUBE3 accelerates proliferation and migration of dental mesenchymal cells via the TGFβ/Smad pathway

**Fig. 2:**
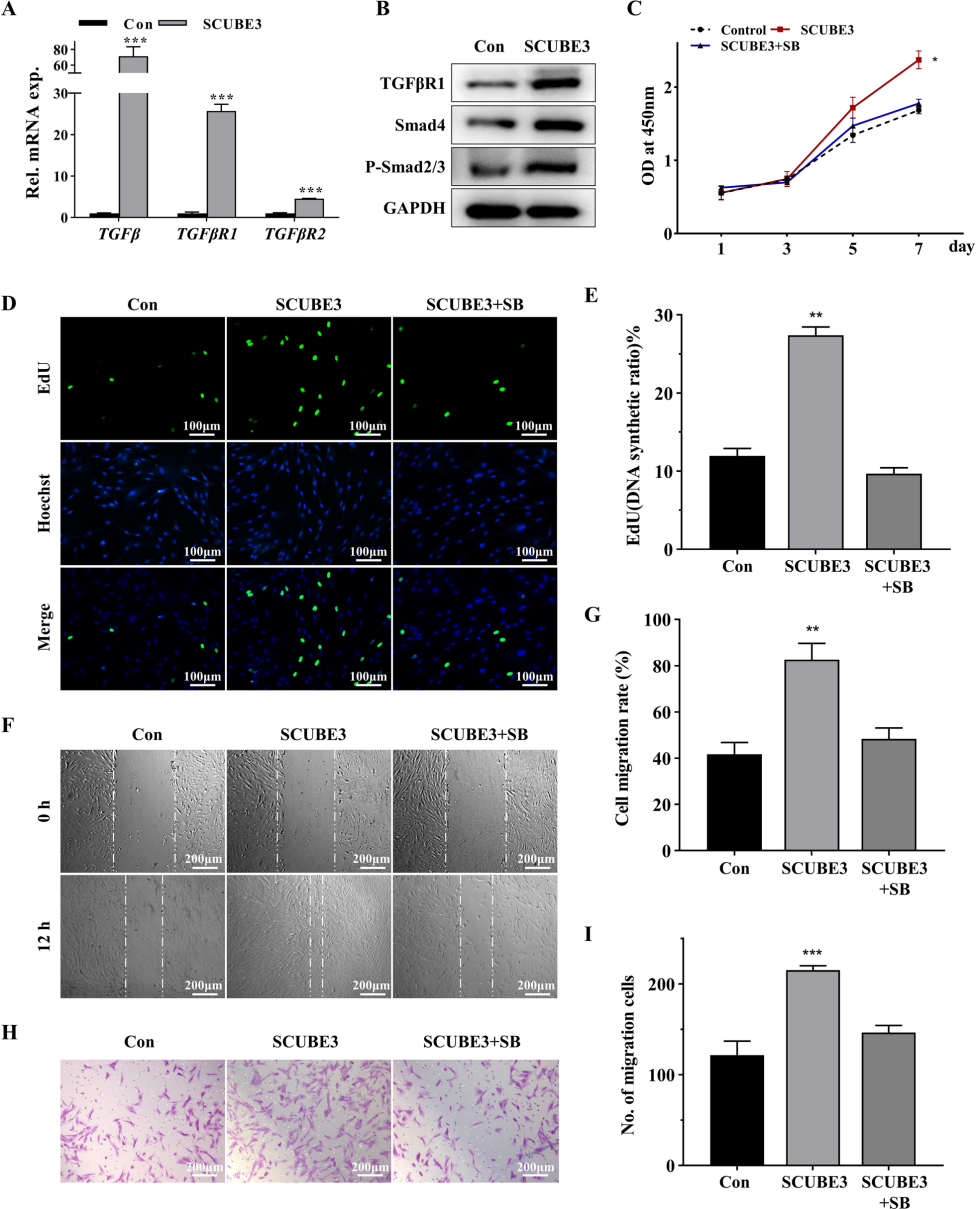
Cell proliferation and migration abilities of hDPSCs treated with exogenous rhSCUBE3. **A** The expression of TGFβ pathway downstream effectors, TGFβ1, TGFβR1, and TGFβR2, was assessed using RT-qPCR. **B** The expression of TGFβ pathway downstream effectors was assessed using Western blot analysis. Full-length blots/gels are presented in Additional file 3. **c** CCK-8 assay. **D, E** EdU assay and quantification. **F, G** Wound healing assay and quantification. **H, I** Transwell migration assay and quantification. *n* = 3 independent biological samples. *P < 0.05, **P < 0.01, ***P < 0.001. SB, SB431542. hDPSC, human dental pulp stem cell

**Fig. 3:**
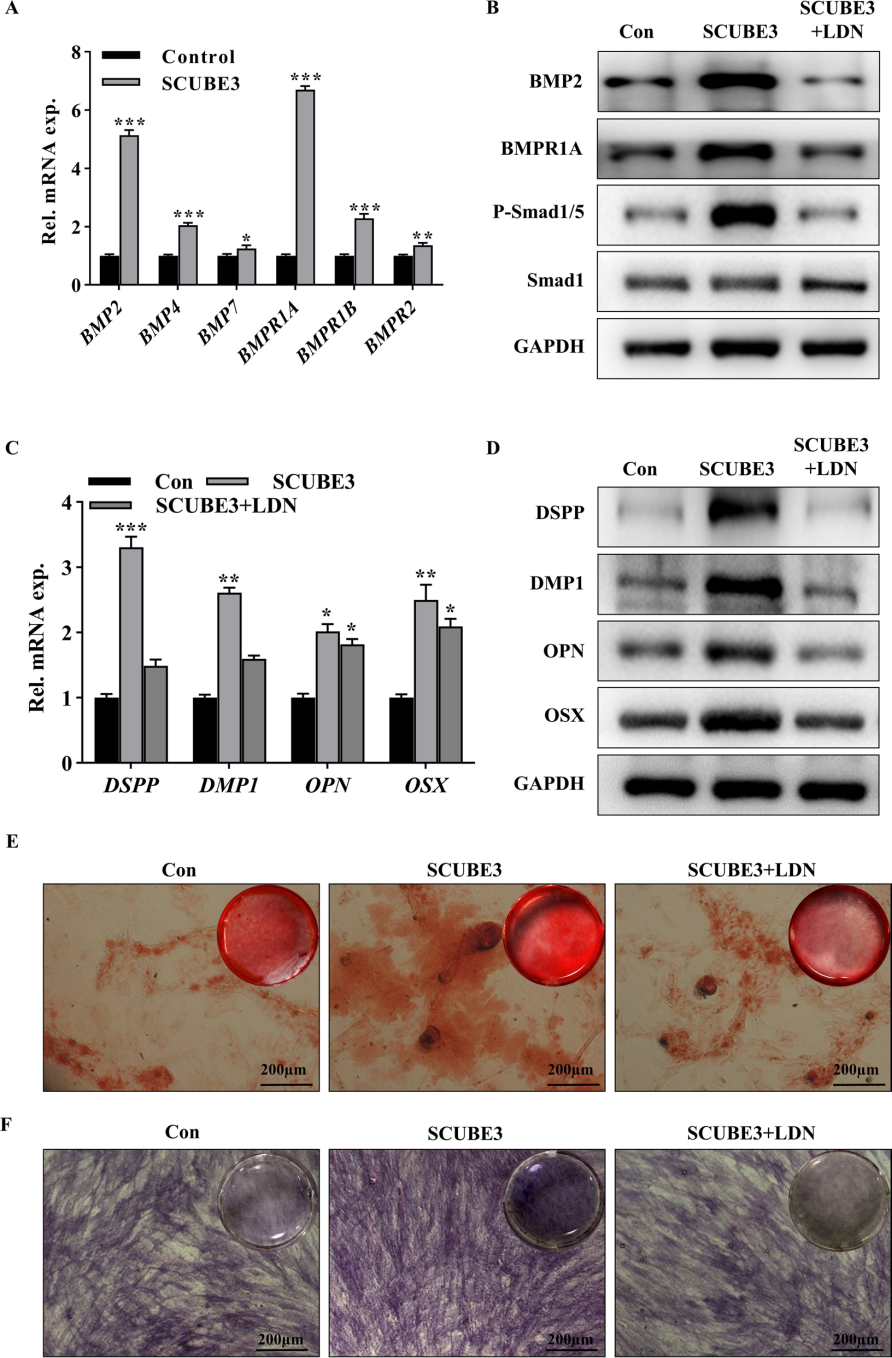
Exogenous SCUBE3 promotes odontoblastic differentiation of hDPSCs via the BMP2/Smad pathway. The cells were exposed to exogenous rhSCUBE3 to explore the differentiation and mechanism of exogenous SCUBE3 in hDPSCs. **A** RT-qPCR was performed to verify the activation of the BMP signalling pathway downstream effectors BMP2, BMP4, BMPR1A, and BMPR1B by SCUBE3. BMP2 and BMPR1A presented the most significant difference relative to the control. **B** HDPSCs were further treated with exogenous rhSCUBE3 or rhSCUBE3 with the BMP2 pathway inhibitor LDN-193189. Western blot analysis was used to detect the BMP signalling pathway downstream effectors BMP2, BMPR1A, Smad1, and p-Smad1/5 expression. Full-length blots/gels are presented in Additional file 3. **C** The expression of the odontoblastic differentiation markers *DSPP*, *DMP1*, *OPN*, and *OSX* in hDPSCs treated with rhSCUBE3 or rhSCUBE3 with LDN-193189 was assessed using RT-qPCR. **D** The expression of the odontoblastic differentiation markers in hDPSCs treated with rhSCUBE3 or rhSCUBE3 with LDN-193189 was assessed using Western blot analyses. Full-length blots/gels are presented in Additional file 3. **E** Alizarin R staining. **F** Alkaline phosphatase activity staining. *n* = 3 independent biological samples. *P < 0.05, **P < 0.01, ***P < 0.001. LDN, LDN-193189

To explore the bio-function of epithelium-derived SCUBE3 in Mes, we used hDPSCs as a Mes model and replaced the epithelium-derived SCUBE3 with rhSCUBE3. CCK-8 (Additional file 2: Fig. S1A) and EdU assays (Additional file 2: Fig. S1B, C) implied increased survival and proliferation ability of rhSCUBE3-treated hDPSCs. In addition, the results of Transwell and wound healing assays showed that exogenous SCUBE3 induced more migrated cells (Additional file 2: Fig. S1D–G). To further study the potential mechanism of SCUBE3 in promoting the proliferation and migration of hDPSCs, in fact, we detected many common pathways, including TGFβ, BMP, PI-3 K-AKT, MAPK signalling pathway, and so on, and found that only TGFβ and BMP signalling pathway were activated in hDPSCs treated with exogenous SCUBE3. Of note, *TGFβ1*, *TGFβR1*, *TGFβR2*, *Smad4*, and *p-Smad2/3* expressions were significantly upregulated (Fig. 2a, b) at the early stage of treatment, suggesting the activation of the TGFβ/Smad signalling pathway. And the BMP signalling pathway was activated at the later stage of treatment (Fig. 3a). Therefore, we speculated that the activation of TGF signalling pathway at the early stage of treatment may be closely related to the proliferation and migration of hDPSCs. To provide further evidence for the mechanism, we exposed hDPSCs to the TGFβ pathway inhibitor SB431542 and found that SB431542 repressed cell growth (Fig. 2c–e) and migration ability (Fig. 2f–i) upregulated by rhSCUBE3. These data verify that SCUBE3 promotes hDPSC proliferation and migration via TGFβ/Smad signalling, and reveal its capacity for cell self-renewal, recruitment of cells, and even the potential for pulp/dentin regeneration.

### Exogenous SCUBE3 is involved in BMP2/Smad pathway-induced odontoblastic differentiation of Mes

In response to epithelium–mesenchyme interactions, *SCUBE3* expression gradually increased in odontoblast-differentiating Mes of the postnatal tooth germ. Therefore, we further explored the temporal relationship between *SCUBE3* and odontoblastic differentiation. Both the mRNA and protein expression of SCUBE3 in hDPSCs treated with OIM positively correlated with that of DSPP (Additional file 2: Fig. S2A, B), revealing that hDPSCs produced endogenous SCUBE3 during odontoblastic differentiation. We then constructed mesenchymal SCUBE3 knockdown using shRNA (Additional file 2: Fig. S3A, B). ShSCUBE3#2-transfected hDPSCs significantly reduced the expression of odontoblastic differentiation-related markers (Additional file 2: Fig. S3C, D), suggesting the *SCUBE3* gene is crucial for odontoblastic differentiation. Meanwhile, exogenous SCUBE3 positively modulated odontoblastic differentiation in a concentration-dependent manner, with 0.5 μg/mL being the optimum concentration (Additional file 2: Fig. S4). RhSCUBE3-treated cells were robustly ARS (Additional file 2: Fig. S3E) and ALP positive (Additional file 2: Fig. S3F). Interestingly, we pre-treated hDPSCs with rhSCUBE3 for 1–6 days and found that exogenous SCUBE3 induced endogenous SCUBE3 expression (Additional file 2: Fig. S5). These data indicate a key role for rhSCUBE3 in modulating odontoblastic differentiation of hDPSCs. To further explore the mechanism of SCUBE3 promoting odontoblastic differentiation of hDPSCs, as *BMP2*, *BMP4*, *BMP7*, *BMPR1A*, *BMPR1B* and *BMPR2* were significantly upregulated at the later stage of treatment (Fig. 3a), suggesting the activation of the BMP/Smad signalling pathway. Treatment with LDN-193189, a BMP signalling inhibitor, counteracted the upregulation of BMP2, BMPR1A, and p-Smad1/5 triggered by rhSCUBE3 (Fig. 3b, Additional file 2: Fig. S6). Furthermore, we speculated that the activation of BMP signalling pathway at the later stage of treatment may be closely related to the odontoblast differentiation of hDPSCs. After inhibiting BMP/Smad signalling, the expression of differentiation-related markers was downregulated (Fig. 3c, d). ARS (Fig. 3e) and ALP (Fig. 3f) staining showed less biomineralization in hDPSCs exposed to LDN-193189. These data indicate that SCUBE3 is involved in the BMP2/Smad pathway-induced odontoblastic differentiation of hDPSCs. Interestingly, we observed that BMP2 was increasingly present (Additional file 2: Fig. S7A) and released into the conditioned medium during differentiation, similar to the expression and secretory pattern of SCUBE3 (Additional file 2: Fig. S7B).

### Exogenous SCUBE3 facilitates odontoblastic differentiation in dentin–pulp organoids

**Fig. 4:**
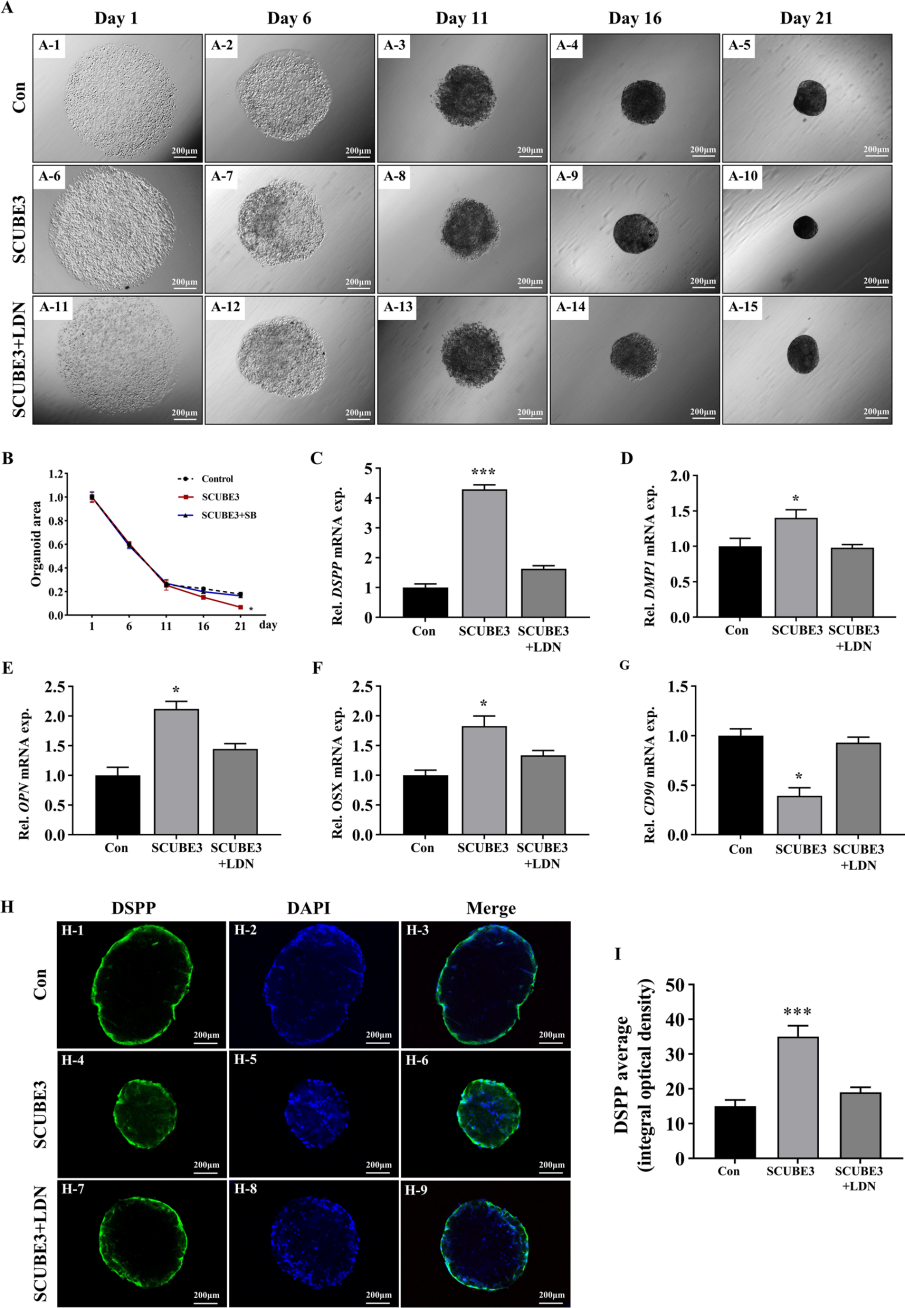
SCUBE3 accelerates the formation and odontoblastic differentiation of dentin–pulp-like organoids. **A** hDPSCs were treated with or without rhSCUBE3 and LDN-193189. The organoids were observed and photographed under a light microscope on days 1, 6, 11, 16, and 21. **B** Quantification of organoid area. **C–F** mRNA expression of the odontoblastic/osteoblastic markers *DSPP*, *DMP1*, *OPN*, and *OSX* in dentin–pulp-like organoids was evaluated using RT-qPCR. **G** mRNA levels of the undifferentiated cell marker CD90 in dentin–pulp-like organoids were evaluated using RT-qPCR. **H, I** Photomicrographs of dentin–pulp-like organoids under confocal immunofluorescence microscopy and quantification with integral optical density. *n* = 3 independent biological samples. *P < 0.05, **P < 0.01, ***P < 0.001

Organoids represent a crucial bridge between 2D cell culture and in vivo animal models [23]. To further verify the effect of SCUBE3 on odontoblastic differentiation, we constructed dentin–pulp organoids with hDPSCs and observed their development using a light microscope. On the first day of culture, hDPSCs from all groups were dispersed (Fig. 4a1, 6, 11) and started to aggregate gradually on day 6 (Fig. 4a2, 7, 12). On day 11, organoids with no difference in any group were exposed to rhSCUBE3 with or without LDN-193189 (Fig. 4a3, 8, 13). HDPSCs began to form condensed spheroids on day 16 (Fig. 4a4, 9, 14). On day 21, faster cell aggregation and more condensed organoid formation were observed in rhSCUBE3-treated organoids than in the other groups (Fig. 4a5, 10, 15). SCUBE3-treated organoids had significantly smaller areas, suggesting that SCUBE3 promotes the development of organoids (Fig. 4b). Additionally, organoid development was repressed in the rhSCUBE3/LDN-193189 group. In the SCUBE3 group, the mRNA expression of *DSPP*, *DMP1*, *OPN*, and *OSX* increased (Fig. 4c–f), while that of *CD90* significantly decreased compared to the control group (Fig. 4g). These results were confirmed using confocal immunostaining for DSPP (Fig. 4h) and integral optical density (Fig. 4i). Collectively, these findings demonstrate that SCUBE3 promotes odontoblastic differentiation in dentin–pulp organoids by activating BMP/Smad signalling.

### Exogenous SCUBE3 promotes dentin–pulp regeneration in vivo

**Fig. 5:**
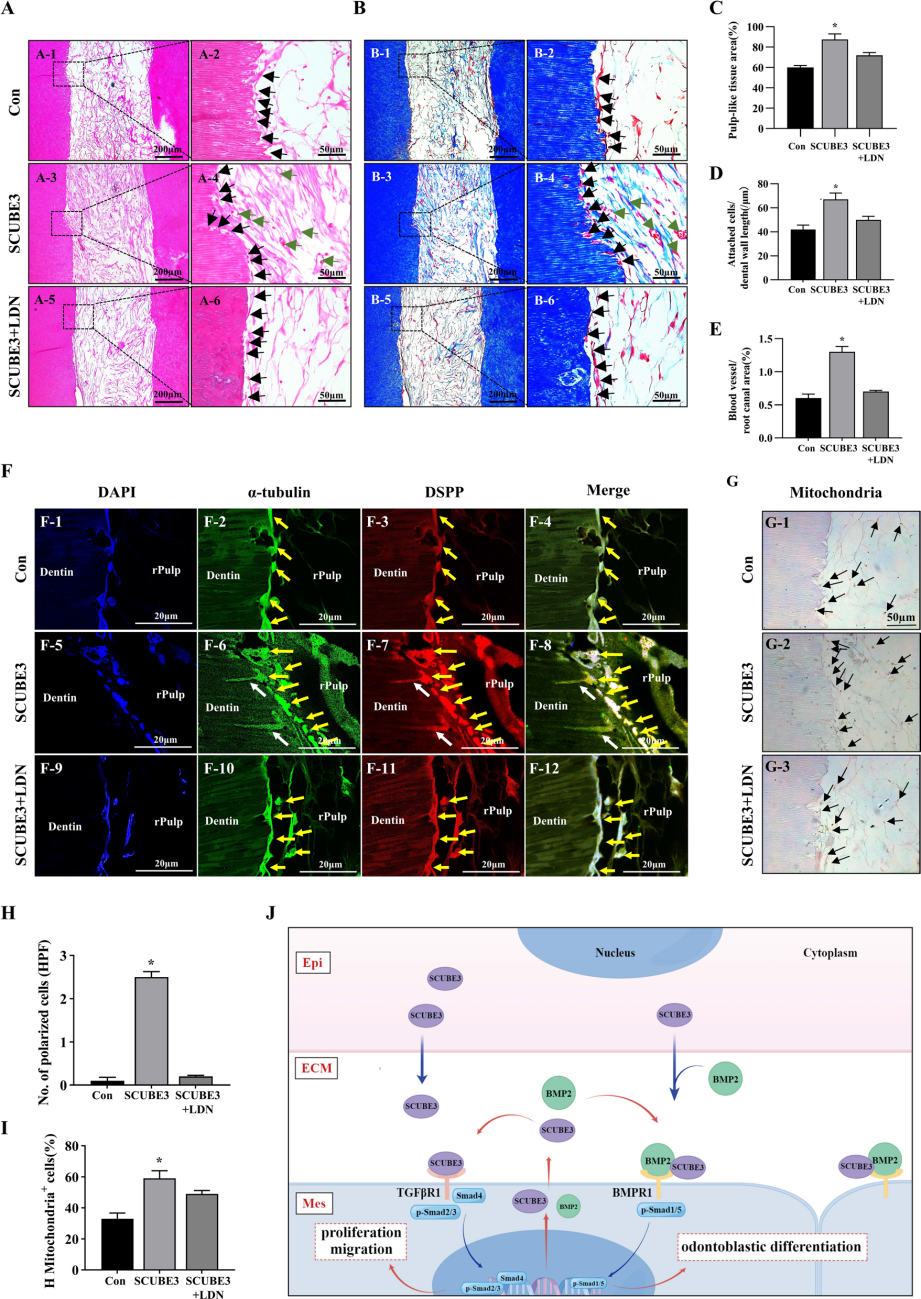
SCUBE3 pre-treatment enhances cell survival, odontoblastic differentiation, cell polarization, and pulp-like tissue formation. **A** Haematoxylin and eosin images of the control, SCUBE3, and SCUBE3 + LDN-193189 groups (A1, 3, 5, respectively), and their higher magnifications (A2, 4, 6). **B** Masson’s trichrome staining images of control, SCUBE3, and SCUBE3 + LDN-193189 groups (B1, 3, 5, respectively), and their higher magnifications (B2, 4, 6, respectively). Black arrowheads show the cells attached to dentin wall. Green arrowheads show new-born blood vessels. **C**–**E** Quantification of new-born pulp-like tissue, attached cell layer, and new-born blood vessels, respectively. **F** Confocal immunofluorescence of α-tubulin and DSPP. Odontoblastic processes with a length of about 20 μm that extended in the dentin tubules were observed in the SCUBE3 pre-treatment group. **G** Human mitochondria immunohistochemistry. Black arrowheads show the human mitochondria-positive cells. **H** Quantification of polarized cells observed in (**F**). **I** Quantification of human mitochondria-positive stained cells. **J** Overall schematic diagram of SCUBE3 in the regulation of odontoblastic differentiation. SCUBE3 is produced and secreted into the ECM by Epi via a paracrine pathway. As a co-receptor of BMP2, secreted SCUBE3 can bind to BMP2 to form a complex. The SCUBE3–BMP2 complex then recruits BMPR1A into raft microdomains, augments the specific interactions between BMP2 and BMPR1A, and activates BMP2/Smad-mediated odontoblastic differentiation in Mes. Additionally, as a ligand of TGFβR1, SCUBE3 can promote cell proliferation and migration via TGF signalling. SCUBE3 also improves endogenous SCUBE3 expression and triggers the autocrine/paracrine secretion of SCUBE3 and BMP2 during differentiation, which can positively promote odontoblastic differentiation of the developing tooth germ in response to epithelium–mesenchyme interactions. *n* = 3 independent biological samples. *P < 0.05, **P < 0.01, ***P < 0.001. rPulp, regenerative pulp. Epi, tooth germ epithelial cells. Mes, tooth germ mesenchymal cells

A semi-orthotropic animal experiment was conducted to further confirm the role of SCUBE3 in promoting vascularized dental pulp regeneration in vivo [24, 25]. After treating hDPSCs with rhSCUBE3 for 1–6 days, the mRNA levels of *SCUBE3* and *DSPP* in hDPSCs were evaluated using RT-qPCR. The hDPSCs pre-treated with rhSCUBE3 for four days demonstrated the highest *SCUBE3* and *DSPP* expression compared to the other groups (Additional file 2: Fig. S4A, B). Subsequently, the decellularized tooth scaffolds were transplanted into the subcutaneous tissue of nude mice under three different conditions: hDPSC-only, hDPSCs pre-treated with rhSCUBE3 for four days, and hDPSCs pre-treated with SCUBE3 and LDN-193189. The harvested root canals in the control group were filled with tissue devoid of enough cells but rich in collagen fibres (Fig. 5a1, 2, b1, 2). The canals of the SCUBE3 group were almost filled with compacted and well-structured connective tissue, along with abundant ECM, collagen fibres, ample cells, and sufficient blood vessels relative to the controls (Fig. 5a3, 4, b3, 4). There was a significant upregulation in the pulp-like tissue filling rate in the SCUBE3 group compared to that in the controls (Fig. 5c), and more attached cell layers (Fig. 5d) and blood vessels (Fig. 5e) were observed in the SCUBE3 group. Surprisingly, DSPP and α-tubulin double stain-positive cell processes that extended in the dentin tubules were observed in the SCUBE3 group (Fig. 5f, h). According to immunohistochemistry results, the cell layer presented human mitochondria (Fig. 5g1–3), revealing that these attached cells were odontoblast-like cells differentiated from hDPSCs. The number of mitochondria-positive cells in the SCUBE3 group was significantly higher than that in the other two groups (Fig. 5i). All these effects of SCUBE3 were reversed by LDN-193189 (Fig. 5a5, 6, b5, 6, f3, g3). Our results indicate that SCUBE3 promoted the survival of transplanted stem cells, the polarization of odontoblast-like cells, and angiogenesis in vivo.

## Discussion

Members of the SCUBE family share a distinct domain organization of at least five recognizable motifs. Previous studies have reported that, during murine odontogenesis, SCUBE1 and SCUBE3 have dynamic reciprocal expressional patterns within the mesenchyme and epithelium, respectively [22]. In contrast, SCUBE2 expression in the developing mouse tooth is negligible [16]. At the late bell stage, the dental epithelium can promote odontoblastic differentiation of undifferentiated ectomesenchymal cells, which entails the interaction of epithelium and mesenchyme via paracrine signalling [26]. The dental epithelium and mesenchyme have been demonstrated to secrete factors that are preferred by reciprocal cells rather than being taken up by themselves and that could evoke differentiation and matrix synthesis [23]. Moreover, epithelium-derived bioactive factors are capable of enhancing odontoblastic differentiation in hDPSCs [27, 28]. It has been reported that the SCUBE3 transcript was strictly localized to the foetal murine tooth germ epithelial tissue [12], and its translation product was a secreted glycoprotein. Therefore, we investigated whether SCUBE3 was involved in epithelium–mesenchyme interactions to affect mesenchymal cell biology.

Several studies using whole-mount transcript identification and section in situ hybridization have confirmed that *Scube3* transcripts strictly localize to the foetal murine tooth germ epithelial tissue [12]. However, the spatiotemporal expression of the SCUBE3 protein during tooth germ development is still unclear. In the present study, our results showed that, in the Epi of prenatal mice, the expression domains and dynamic changes in SCUBE3 protein were consistent with those of *SCUBE3* mRNA. In postnatal mice, the expression of SCUBE3 in Epi decreased significantly. In tooth germ Mes, low SCUBE3 protein expression was found in the ECM of mesenchymal tissue at E14.5 using immunohistochemistry staining. At E16.5, the expression of SCUBE3 in the Mes started to become detectable and increased gradually in the differentiating odontoblasts of postnatal tooth germ. Obviously, the SCUBE3 protein can be detected earlier than the *SCUBE3* transcript in Mes during tooth development, suggesting that the SCUBE3 protein in the Mes of prenatal tooth germ may derive from the Epi. Notably, a co-culture system of tooth germ Epi and Mes was constructed and convincingly confirmed that the epithelium-derived SCUBE3 can translocate to Mes via epithelium–mesenchyme interactions.

Subsequently, we explored the bio-function of epithelium-derived SCUBE3 on mesenchymal cells via epithelial–mesenchymal interactions. HDPSCs were used as the model of dental mesenchymal cells and rhSCUBE3 replaced the epithelium-derived SCUBE3 protein. CCK-8 and EdU assays showed that rhSCUBE3 promoted the proliferation of hDPSCs. Transwell and wound healing assays both verified the capacity of rhSCUBE3 to accelerate the migration rate of hDPSCs. Former studies have shown that the knockdown of *SCUBE3* suppressed lung cancer invasion and metastasis [29] and breast cancer cell growth, invasion, and migration [30]*.* Our findings demonstrate that exogenous SCUBE3 also enhances cell proliferation and migration of dental Mes.

Meanwhile, the presence of rhSCUBE3 resulted in the upregulation of DSPP and DMP1 expression in hDPSCs, demonstrating that the application of exogenous SCUBE3 can help promote odontoblastic differentiation of dental Mes. Interestingly, even without rhSCUBE3 stimulation, the changes in both SCUBE3 mRNA and protein levels were consistent with the increase in DSPP expression during the differentiation of hDPSCs, revealing that odontoblasts themselves can generate SCUBE3 protein via autocrine signalling during odontoblastic differentiation. We used shRNA to knockdown the endogenous *SCUBE3* expression, and both *DSPP* and *DMP1* were downregulated. These results thus demonstrate that *SCUBE3* is a vital gene to modulate the odontoblastic differentiation of dental Mes. Notably, exogenous rhSCUBE3 can partly rescue the suppressed odontoblastic differentiation of the cells transfected with shRNA, suggesting that exogenous SCUBE3 could also enhance endogenous SCUBE3 expression and trigger SCUBE3 autocrine/paracrine secretion in differentiating odontoblasts, similar to BMP2 [30]. Since the SCUBE3 protein appears earlier than the *SCUBE3* transcript in Mes, we speculate that the translocation of epithelium-derived SCUBE3 to the mesenchyme may be involved in the induction of odontoblastic differentiation. This assumption requires further evaluation using additional lines of evidence, such as epithelial cell-specific knockout mouse models.

The specific CUB-like structure of SCUBE3 can bind to various signalling receptors, including BMPR, TGF-βR, and VEGF, acting as a ligand or co-receptor to trigger a series of biological effects [31–33]. For instance, both the SCUBE3 protein and the C-terminal CUB domain fragment can bind to the TGF-β type II receptor through the C-terminal CUB domain, activating TGF-β signalling and triggering the epithelial–mesenchymal transition, deposition of the ECM, invasion into adjacent tissues, and angiogenesis [34]. The TGF-β, BMP, canonical WNT/β-catenin, and MAPK signalling pathways play critical roles in the odontoblastic differentiation of dental Mes [35, 36]. Based on the suggested dual role of BMP/TGF-β as a downstream activator of SCUBE3 and as an essential pathway regulating odontoblastic differentiation [34, 37], we hypothesized that SCUBE3 probably exerted effects on the BMP/TGF-β signalling pathway as an auxiliary modulator. Treating hDPSCs with exogenous SCUBE3 led to the activation of the TGF-β and BMP pathways. According to our results, after the TGF-β signalling was suppressed by SB431542, the proliferation and migration potentials were downregulated, while there was no significant change in differentiation ability. These findings suggested that TGF-β signalling mainly modulates the proliferation and migration of dental Mes treated with rhSCUBE3. However, treatment with the BMP signalling inhibitor LDN-193189 not only downregulated the expression of BMP signalling pathway downstream effectors, BMPR1A and *p*-Smad1/5, but also inhibited the expression of odontoblast markers, DSPP and DMP1, and osteoblast markers, OPN and OSX, demonstrating that this pathway is essential for SCUBE3-induced odontoblastic differentiation. These results were further verified using dentin–pulp-like organoids. It has been reported that SCUBE3 acts as a BMP2/BMP4 auxiliary receptor and positively modulates signalling by enhancing the specific interactions between BMPs and BMP receptors [15]. Our results demonstrated that SCUBE3 is involved in the BMP-dependent odontoblastic differentiation process of hDPSCs. Furthermore, exogenous SCUBE3 not only promoted the upregulation of odontoblast differentiation-related markers, but also induced the production and secretion of SCUBE3 and BMP2. These results suggest that exogenous SCUBE3 could trigger autocrine/paracrine signalling of SCUBE3 during the differentiation of hDPSCs, constantly promoting odontoblastic differentiation in the developing tooth germ via epithelium–mesenchyme interactions.

Semi-orthotopic models using subcutaneous tooth fragment implantation have been widely used to investigate pulp–dentin regeneration [20, 24]. We used an animal model to study the clinical application of SCUBE3. In the SCUBE3 pre-treatment group, we observed well-organized pulp-like tissues and more human mitochondrial-positive cells, along with many DSPP- and α-tubulin-positive odontoblast-like cells adhered to the dentin walls. The cell layers presented odontoblastic processes that extended in the dentin tubules. These results suggested that SCUBE3 pre-treatment promotes transplanted hDPSC odontoblastic differentiation with polarization of odontoblast-like cells. Nevertheless, pre-dentin was not detected in any of the groups, which may be ascribed to the relatively short observation time. Surprisingly, although rich new-born blood vessels were observed in the rhSCUBE3-pre-treated group, the vessels showed negative staining for human mitochondria, indicating that the new-born vasculature was formed by the host cells. Moreover, hDPSCs have little capacity to switch to a vascular endothelial phenotype when transplanted into murine hosts [38, 39]. The abundant regenerated vasculature suggests that SCUBE3 pre-treatment might promote angiogenesis by regulating the migration of vascular endothelial cells from the host. However, the mechanism of cell polarization and angiogenesis regulated by SCUBE3 still needs further exploration in the future.

## Conclusions

Our results confirmed that SCUBE3 protein expression shows a dynamic temporospatial distribution pattern during dentin genesis in the developing tooth germ, which is inconsistent with transcript expression. SCUBE3 translocated from the tooth germ epithelium to the mesenchyme via epithelium–mesenchyme interactions. Additionally, it was confirmed that SCUBE3 accelerates proliferation and migration of hDPSCs via the TGFβ/Smad pathway. Furthermore, in in vitro and organoid models, SCUBE3 demonstrated strong potential for inducing odontoblastic differentiation of hDPSCs via the BMP2/Smad pathway (Fig. 5j). Its capacity to promote vascularized pulp regeneration, including transplanted cell survival, odontoblastic differentiation, cell polarization, and new-born vessels in vivo, was verified using a semi-orthotopic model. These results confirm that finding cues from tooth development is a sound approach for developing novel strategies for pulp–dentin regeneration. To the best of our knowledge, the present study is the first to establish that epithelium-derived SCUBE3 can function as a potential growth factor for dental pulp regeneration and angiogenesis and the first to verify that *SCUBE3* is a vital gene to modulate odontoblastic differentiation of mesenchymal cells. Our findings also provide a crucial theoretical framework for the use of SCUBE3 to promote dentin–pulp regeneration.

## Supplementary Information

**Additional file 1**. Supplementary tables.

**Additional file 2**. Supplementary figures.

**Additional file 3**. Supplementary figures of full-length blots/gels.

## Data Availability

The datasets supporting the conclusions of this article are included within the article and its additional files.

## Abbreviations

ALP: Alkaline phosphatase
ANOVA: Analysis of variance
ARS: Alizarin red
E: Embryonic day
ECM: Extracellular matrix
EdU: 5-Ethynyl-2′-deoxyuridine
Epi: Epithelial cells
FBS: Foetal bovine serum
hDPSCs: Human dental pulp stem cells
mEpi: Mouse tooth germ epithelial cells
Mes: Mesenchymal cells
mMes: Mouse tooth germ mesenchymal cells
NC: Negative control
OIM: Osteogenic inductive medium
P: Postnatal day
PBS: Phosphate-buffered saline
RT-qPCR: Quantitative reverse transcription polymerase chain reaction
SCUBE3: Signal peptide-CUB-EGF domain-containing protein 3
SD: Standard deviation
shRNA: Short hairpin RNA
