# Supplementary material for: Epithelium-derived SCUBE3 promotes polarized odontoblastic differentiation of dental mesenchymal stem cells and pulp regeneration
Source: Stem Cell Res Ther. 2023 May 15;14:130. doi: 10.1186/s13287-023-03353-0 (PMC10186660; doi:10.1186/s13287-023-03353-0)
Supplement: Supplementary file 1 — Additional file 1. Supplementary tables. [file 13287_2023_3353_MOESM1_ESM.doc]

**Epithelium-derived SCUBE3 promotes polarized-odontoblast differentiation of dental mesenchymal stem cells and pulp regeneration**

**Table S1.** Sequences of shRNA used in this study.

| shRNA | shRNA sequence (5' - 3') |
| --- | --- |
| *shCon*  *shSCUBE3-1*  *shSCUBE3-2*  *shSCUBE3-3* | CCGGTTCTCCGAACGTGTCACGTTTCAAGAGAACGTGACACGTTCGGAGAATTTTTG  CACCGCATCTGCAAGTCTGGCTACACTCGAGTGTAGCCAGACTTGCAGATGC  CACCGCAGTTGCAAGAAAGGCTATACTCGAGTATAGCCTTTCTTGCAACTGC  CACCGCTACCTGTTGTATGGTATCACTCGAGTGATACCATACAACAGGTAGC |

**Table S2. RT-qPCR primer sequences.**

| Species | Genes | Primer sequence (5' - 3') |
| --- | --- | --- |
| Human  Mouse | *GAPDH*  *GAPDH*  *DSPP*  *DSPP*  *DMP1 DMP1*  *OPN*  *OPN*  *OSX*  *OSX*  *CD90*  *CD90*  *SCUBE3*  *SCUBE3*  *BMP2*  *BMP2*  *BMP4*  *BMP4*  *BMP7*  *BMP7*  *BMPR1A*  *BMPR1A*  *BMPR1B*  *BMPR1B*  *TGFβ1*  *TGFβ1*  *TGFβR1*  *TGFβR1*  *TGFβR2*  *TGFβR2*  *Gapdh*  *Gapdh*  *Scube3*  *Scube3* | Forward primer 5’ -GGAGCGAGATCCCTCCAAAAT-3’  Reverse primer 5’ -GGCTGTTGTCATACTTCTCATGG -3’  Forward primer 5’ -CTAAGTGGGCAGAAGTAGGAGGGA-3’  Reverse primer 5’ -TGTTGCTTCTGTTTGTGGCTCC-3’  Forward primer 5’ -TTCCTCTTTGAGAACATCAACCTG-3’  Reverse primer 5’ -ACTCACTGCTCTCCAAGGGT -3’  Forward primer 5’ -GGAGTTGAATGGTGCATACAAGG-3’  Reverse primer 5’ -CCACGGCTGTCCCAATCAG-3’  Forward primer 5’ -CTGTGAAACCTCAAGTCCTATGGA-3’  Reverse primer 5’ -GCTCTGCAGTCAAGGGAGATG-3’  Forward primer 5’ -CAGCATCGCTCTCCTGCTAA-3’  Reverse primer 5’ -ACTGGATGGGTGAACTGCTG-3’  Forward primer 5’ -ATGCCGTCCTGGCTTTGAG-3’  Reverse primer 5’ -CCGTCGGTATGGAGCACAAA-3’  Forward primer 5’ -CTCTGACGAGGTCCTGAGCGAGTTC-3’  Reverse primer 5’ -GGCTGACCTGAGTGCCTGCGATACA-3’  Forward primer 5’ -TGAGCCTTTCCAGCAAGTTT-3’  Reverse primer 5’ -CTTCCCCGTCTCAGGTATCA-3’  Forward primer 5’ -GGCAGGACTGGATCATCG-3’  Reverse primer 5’ -AAGTGGACCAGCGTCTGC-3’  Forward primer 5’ -TCAGCGAACTATTGCCAAACAG-3’  Reverse primer 5’ -ACGCCATTTACCCATCCACA-3’  Forward primer 5’ -TGATGGACCTATACACCACAGG-3’  Reverse primer 5’ -ATAGTCCTTTGGACCAGCAGAG-3’  Forward primer 5’ -CGCATCCTAGACCCTTTCTCCTC-3’  Reverse primer 5’ -GGTGTCTCAGTATCCCACGGAAAT-3’  Forward primer 5’ -GGAGAAGTTTGGAGAGGAAAGT-3’  Reverse primer 5’ -ACCAGAGCTGAGTCCAAGTACC-3’  Forward primer 5’ -GTAGCTCTGATGAGTGCAATGAC-3’  Reverse primer 5’ -CAGATATGGCAACTCCCAGTG-3’  Forward primer 5’ -GTGGCAAAGTGGAGATTGTTGCC-3’  Reverse primer 5’ -TGTGCCGTTGAATTTGCCGT-3’  Forward primer 5’ -TTCACTGGAACGGGAAGGATTG-3’  Reverse primer 5’ -GGACAGGTCAGGGCACAAGTA-3’ |
