# Supplementary material for: Epithelium-derived SCUBE3 promotes polarized odontoblastic differentiation of dental mesenchymal stem cells and pulp regeneration
Source: Stem Cell Res Ther. 2023 May 15;14:130. doi: 10.1186/s13287-023-03353-0 (PMC10186660; doi:10.1186/s13287-023-03353-0)
Supplement: Supplementary file 2 — Additional file 2. Supplementary figures. [file 13287_2023_3353_MOESM2_ESM.doc]

**Epithelium-derived SCUBE3 promotes polarized-odontoblastic differentiation of dental mesenchymal stem cells and pulp regeneration**


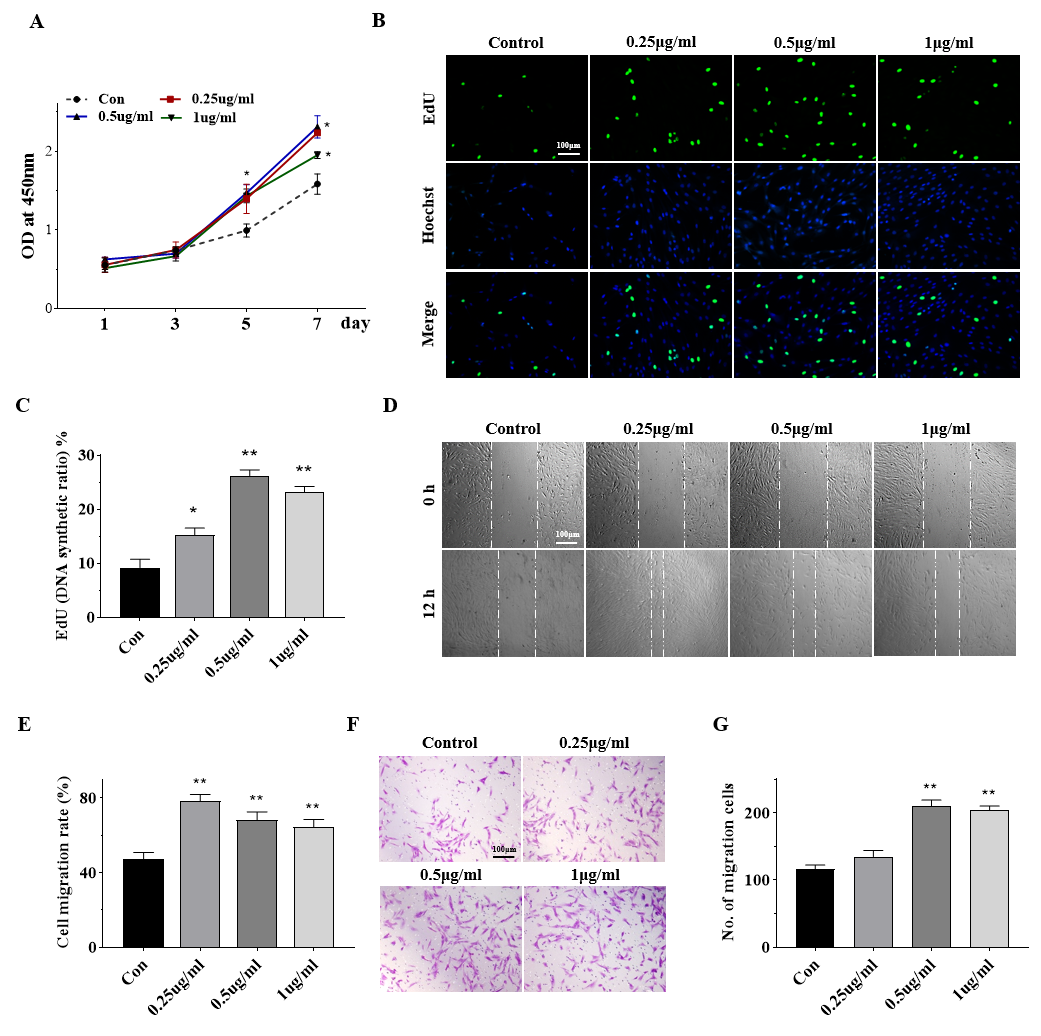
**Supplementary Fig. 1.** rhSCUBE3 stimulated cell proliferation and migration of hDPSCs in a concentration-dependent manner. HDPSCs were exposed to 0.25, 0.5 and 1μg/ml rhSCUBE3 protein. CCK-8 (**A**) and EdU (**B, C**) assays showed that rhSCUBE3 had role in upregulating proliferation in hDPSCs. 0.25~0.5μg/ml rhSCUBE3 presented the highest cell growth rate. Scale bars: 100 µm. Wound healing

implied rhSCUBE3 promoted cell migration with 0.25~0.5μg/ml rhSCUBE3 being the most effective concentration range. n = 3 independent biological samples. **P* < 0.05, ***P* < 0.01, ****P* < 0.001. hDPSCs, human dental pulp stem cells.


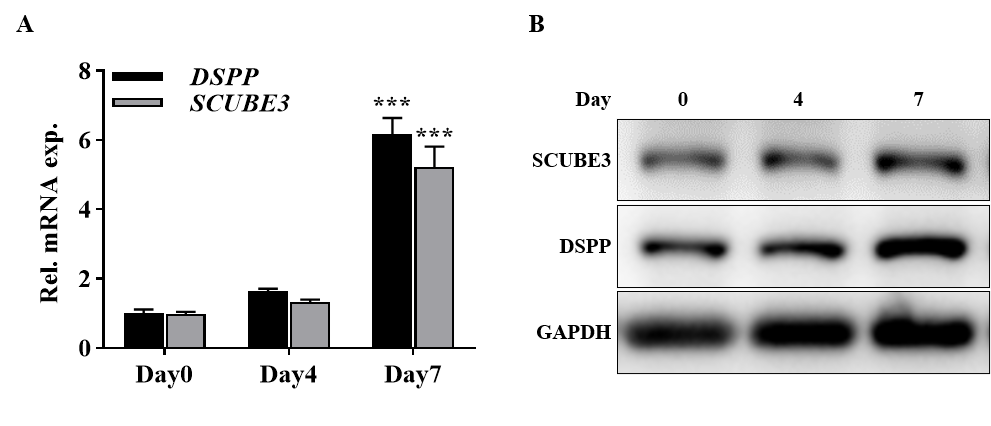
 **Supplementary Fig. 2** SCUBE3 expression is elevated in dental mesenchymal stem cells undergoing odontoblastic differentiation. (**A**) Levels of *SCUBE3* and *DSPP* gene at different induction time points of odontoblastic differentiation in hDPSCs were evaluated by RT-qPCR. (**B**) The protein levels of SCUBE3 and DSPP were detected by Western blot analysis. SCUBE3 gene and protein expression levels were both significantly elevated in hDPSCs after 7 d of being cultured in osteogenic inductive medium. n = 3 independent biological samples. **P < 0.05, **P < 0.01, ***P < 0.001.* hDPSCs, human dental pulp stem cells.


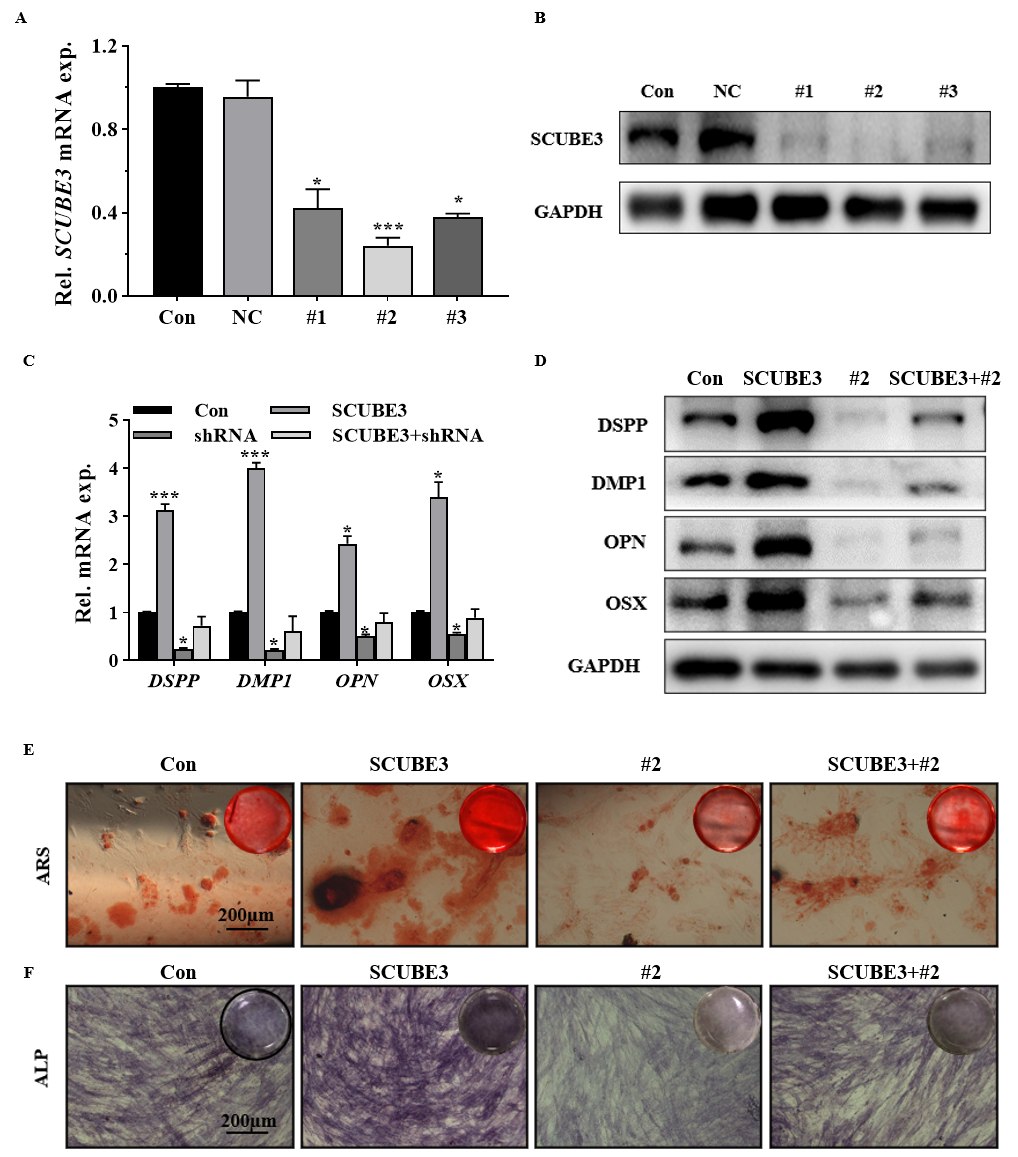
**Supplementary Fig. 3** *SCUBE3* was a vital gene regulating odontoblastic differentiation of dental mesenchymal stem cells. To confirm shRNA silencing efficiency, RT-qPCR (**A**) and Western blot analysis (**B**) were performed on hDPSCs transfected with either control or shRNAs. SCUBE3 is expressed in hDPSCs transfected with control shRNA, whereas its expression is drastically reduced in hDPSCs transfected with SCUBE3 shRNAs, with shSCUBE3#2 being the most effective shRNA. hDPSCs were transfected with control shRNA or SCUBE3 shRNA#2 and with or without rhSCUBE3. (**C**) Expression levels of odontoblastic differentiation markers, including *DSPP*, *DMP1*, *OPN*, and *OSX* mRNA, were analyzed using RT-qPCR. (**D**) The protein levels of odontoblastic differentiation markers in hDPSCs were evaluated by western blot. (**E**) Alizarin Red staining of hDPSCs. (**F**) Alkaline phosphatase activity staining of hDPSCs. Results are expressed as relative expression to GAPDH. Data are presented as mean ± SEM of 3 independent experiments. n = 3 independent biological samples. **P < 0.05, **P < 0.01, ***P < 0.001.* hDPSCs, human dental pulp stem cells.


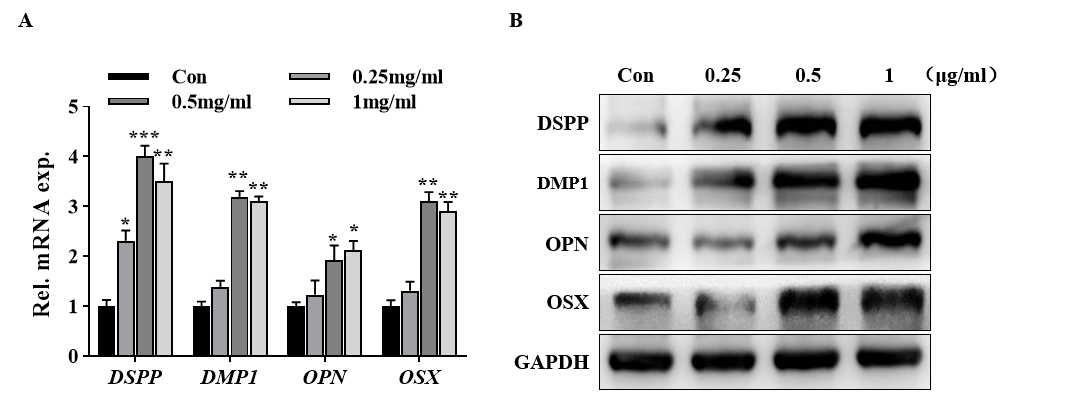
**Supplementary Fig. 4** Exogenous SCUBE3 positively modulated odontoblastic differentiation of hDPSCs in a concentration-dependent manner with 0.5 μg/ml being the optimum concentration. hDPSCs were cultured in OIM add with 0, 0.25, 0.5, and 1 μg/ml rhSCUBE3 for 7 d. RT-qPCR (**A**) and Western blot analysis (**B**) were performed to evaluated the expression levels of odontoblastic differentiation markers in hDPSCs. n = 3 independent biological samples. **P < 0.05, **P < 0.01, ***P < 0.001.* hDPSCs, human dental pulp stem cells.


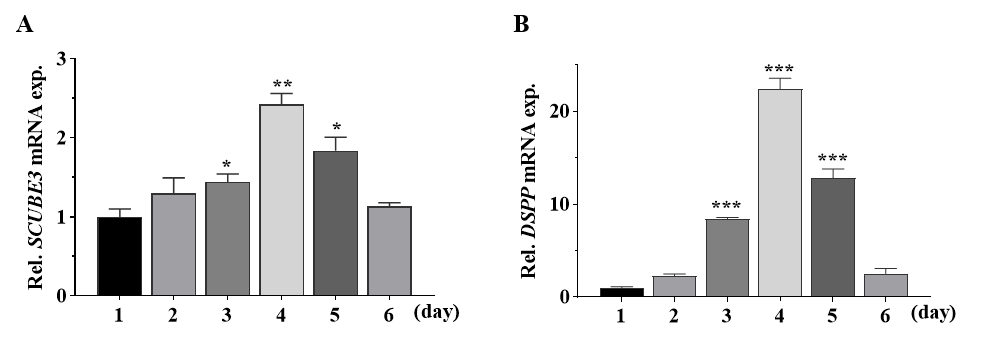


**Supplementary Fig. 5** Exogenous SCUBE3 highly modulated endogenous SCUBE3 and DSPP expression. hDPSCs were cultured in osteogenic inductive medium with 0.5 μg/ml rhSCUBE3. (**A, B**) The SCUBE3 and DSPP gene expression in hDPSCs was evaluated using RT-qPCR. Both SCUBE3 and DSPP mRNA levels significantly increased since 3 d and raised to peak at 4 d. After 5 d, the SCUBE3 and DSPP mRNA levels detected were gradually reduced but remained stronger relative to 1 d. n = 3 independent biological samples. **P < 0.05, **P < 0.01, ***P < 0.001.* hDPSCs, human dental pulp stem cells.

**Supplementary Fig. 6** Western blotting was conducted to analyze the optimum concentration of LDN-193189, the BMP signaling pathway inhibitor at the protein level. n = 3 independent biological samples.


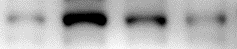


**SCUBE3**

**100**

**0**

**50**

**0**

**LDN-193189**

**p-Smad1/5**

**Smad1**


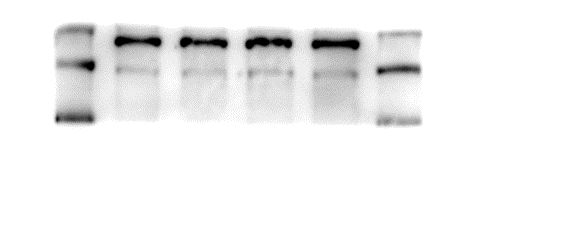

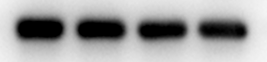


**GAPDH**

**(ng/ml)**


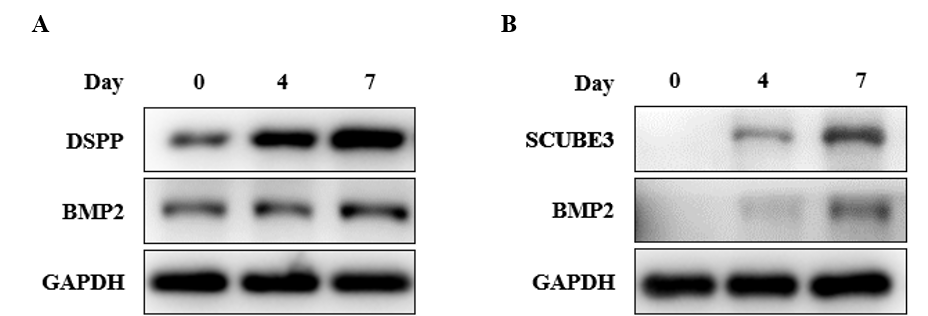
**Supplementary Fig. 7** BMP2 was increasingly expressed and released into the conditioned medium during odontoblastic differentiation of hDPSCs. (**A**) BMP2 levels in whole-cell lysates of hDPSCs on the indicated days during odontoblastic differentiation were evaluated by western blot. (**B**) Western blot of secretory SCUBE3 and BMP2 protein in hDPSCs culture medium during odontoblastic differentiation. n = 3 independent biological samples. hDPSCs, human dental pulp stem cells.
