# Supplementary material for: Epithelium-derived SCUBE3 promotes polarized odontoblastic differentiation of dental mesenchymal stem cells and pulp regeneration
Source: Stem Cell Res Ther. 2023 May 15;14:130. doi: 10.1186/s13287-023-03353-0 (PMC10186660; doi:10.1186/s13287-023-03353-0)
Supplement: Supplementary file 3 — Additional file 3. Supplementary figures of full-length blots/gels. [file 13287_2023_3353_MOESM3_ESM.pdf]

Supplementary file 3

full-length blots/gels

Figure 1F

SCUBE3

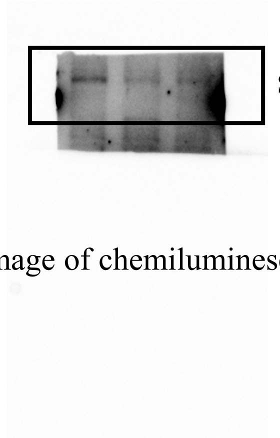

Image of chemiluminescence

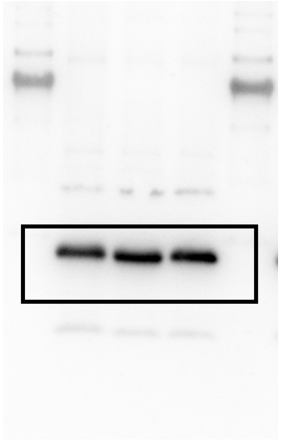

Image of chemiluminescence

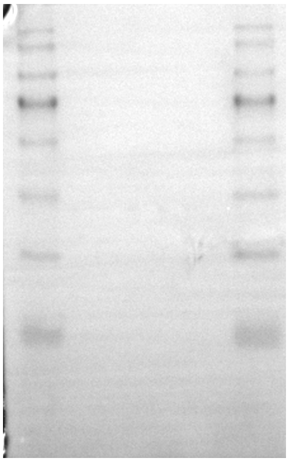

Image of white light

AMBN

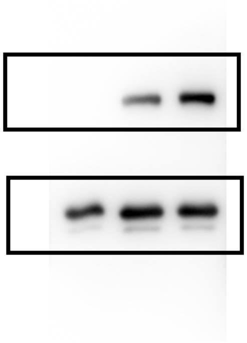

Image of chemiluminescence

Figure 1F. Full-length blots/gels of SCUBE3 and AMBN expression in whole-cell lysates of LS-8 cells on the indicated days of ameloblastic differentiation. Boxes areas are the samples we labelled.

Figure 1G

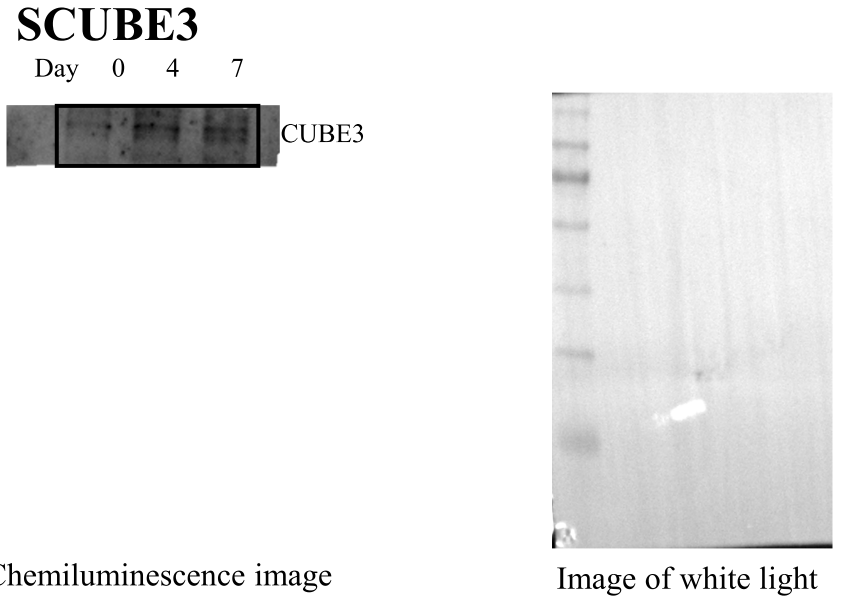

Figure 1G. Full-length blots/gels of secretory SCUBE3 in the conditioned medium of LS-8 during ameloblastic differentiation for 0, 4, 7 days. Boxes areas are the samples we labelled.

Figure 1H

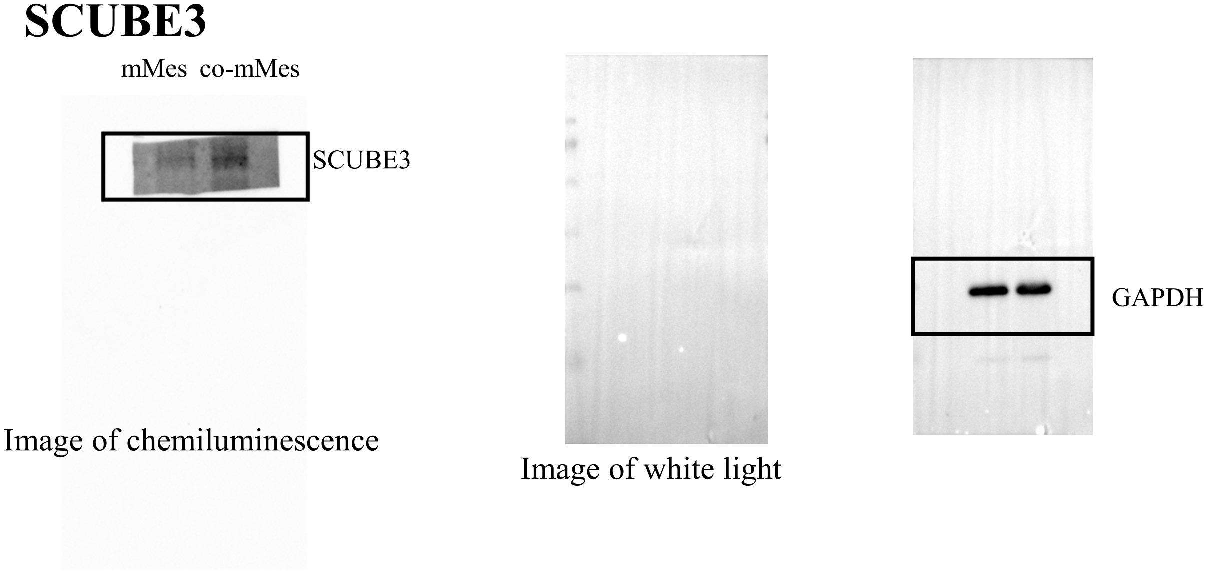

Figure 1H. Full-length blots/gels of SCUBE3 protein in the untreated mMes and co-cultured mMes. Boxes areas are the samples we labelled.

Figure 2 B

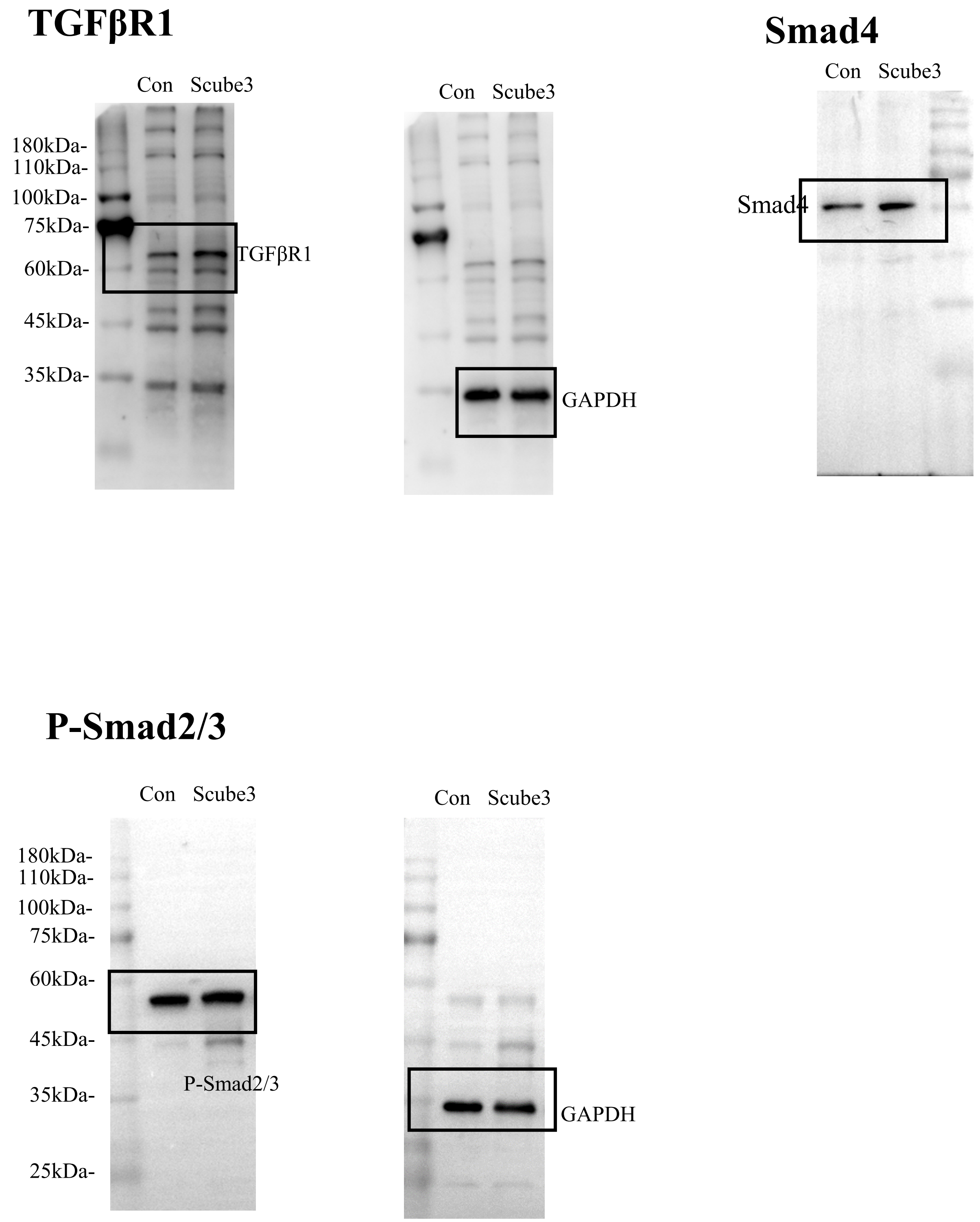

Figure 2B. Full-length blots/gels of TGFβ pathway downstream effectors (TGFβR1, Smad4 and P-Smad2/3) of cells treated with of without SCUBE3. Boxes areas are the samples we labelled.

Figure 3 B

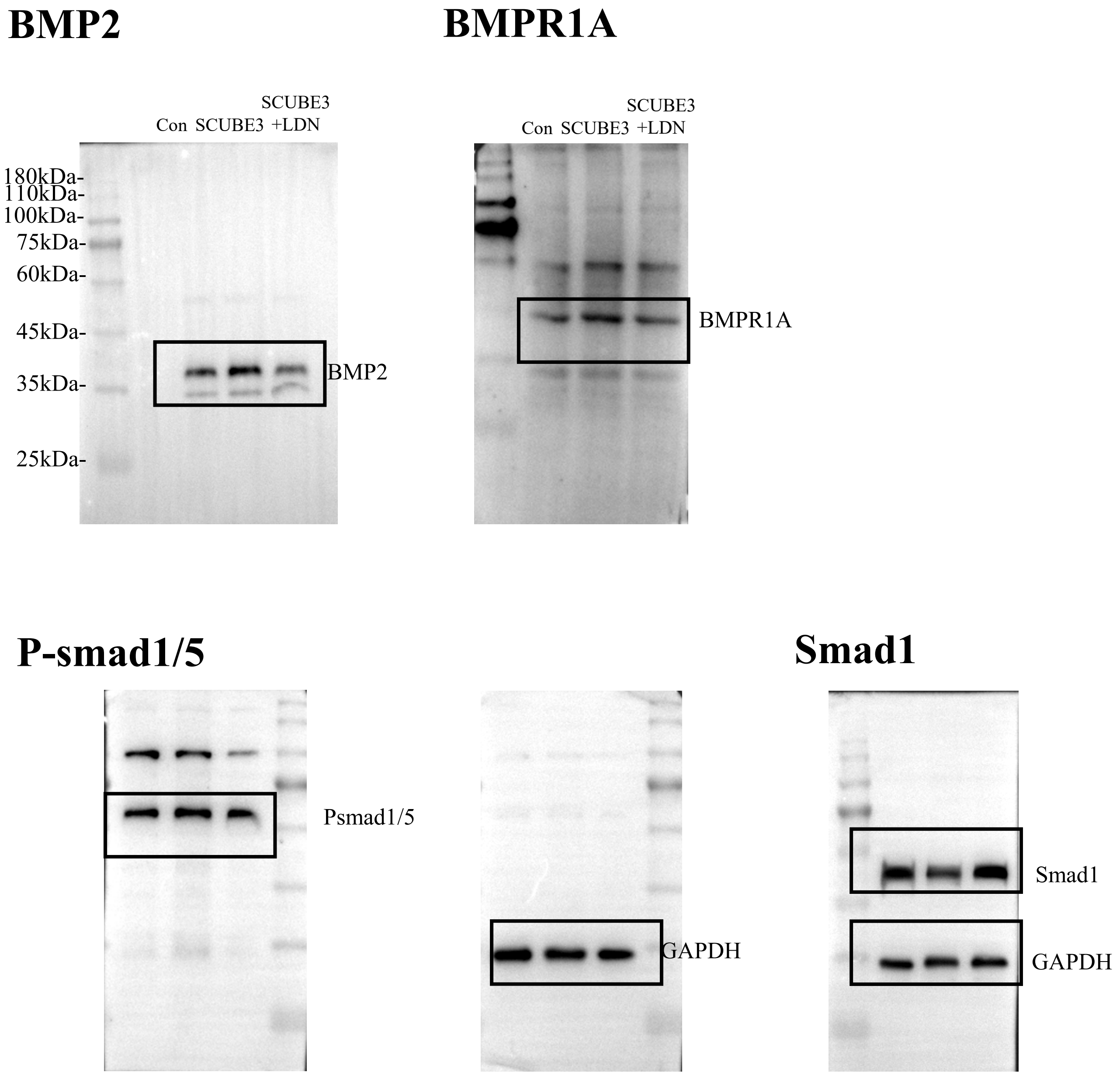

Figure 3B. HDPSCs were further treated with exogenous rhSCUBE3 or rhSCUBE3 with the BMP2 pathway inhibitor LDN-193189. Full-length blots/gels of the BMP signalling pathway downstream effectors BMP2, BMPR1A, Smad1, and p-Smad1/5 expression were presented. Boxes areas are the samples we labelled.

Figure 3 D

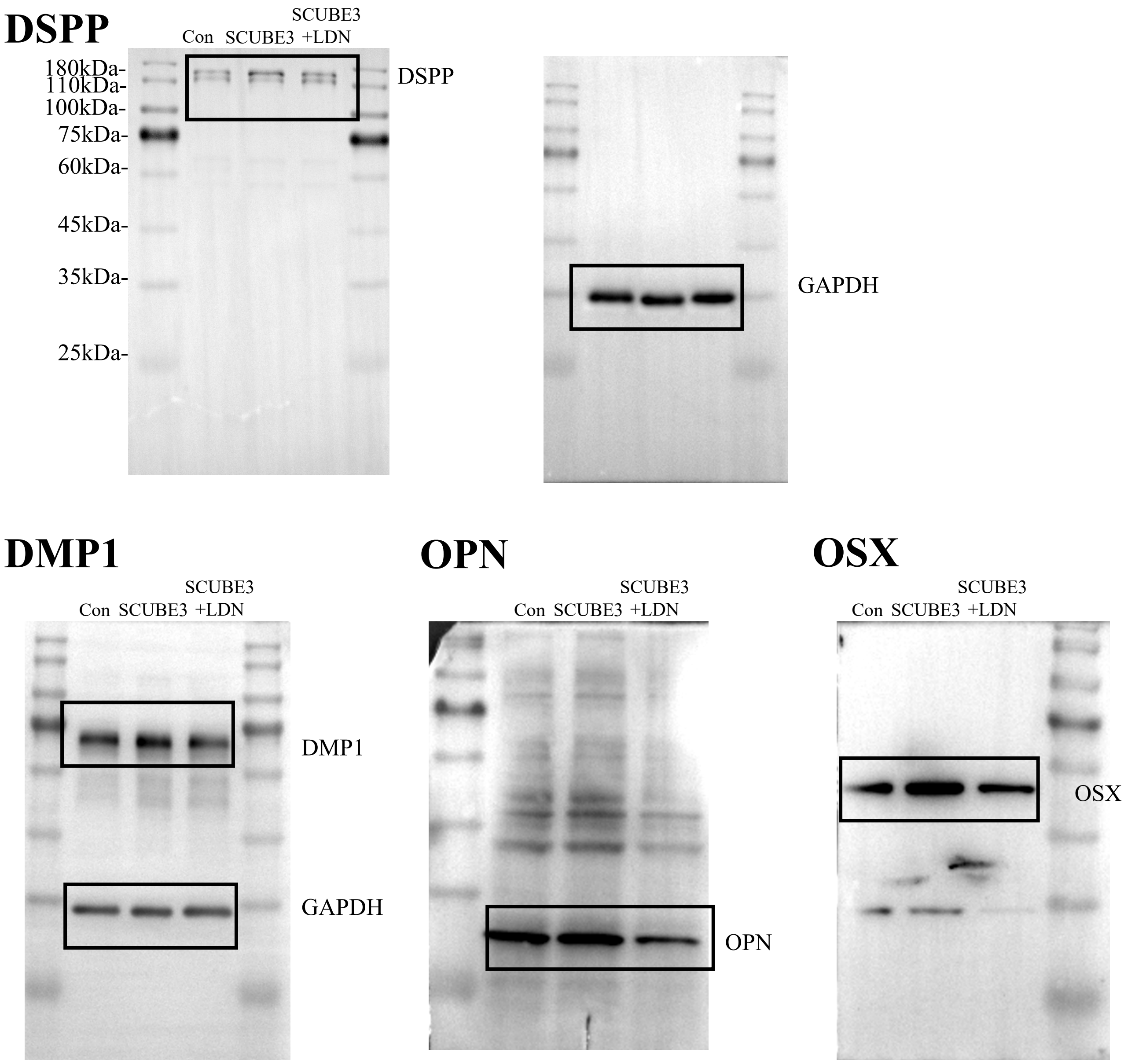

Figure 3D. The expression of the odontoblastic differentiation markers in hDPSCs treated with rhSCUBE3 or rhSCUBE3 with LDN-193189 was assessed using western blot analyses. Full-length blots/gels of the DSPP, DMP1, OPN, and OSX expression were presented. Boxes areas are the samples we labelled.

Supplementary Fig. 2B

SCUBE3

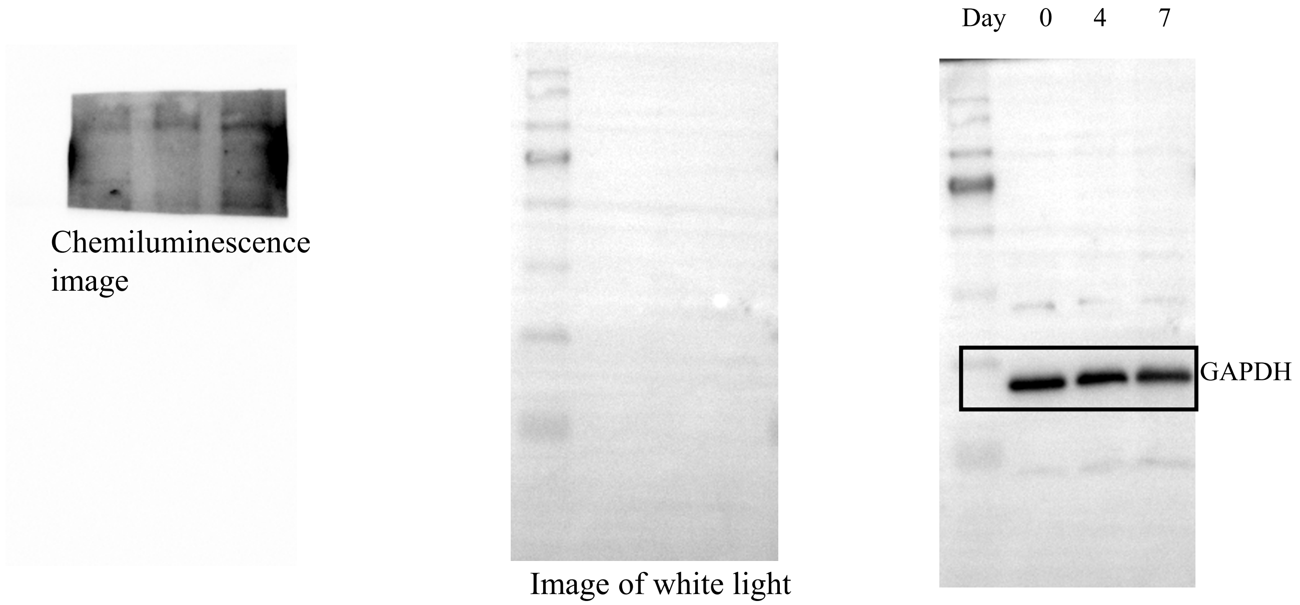

DSPP

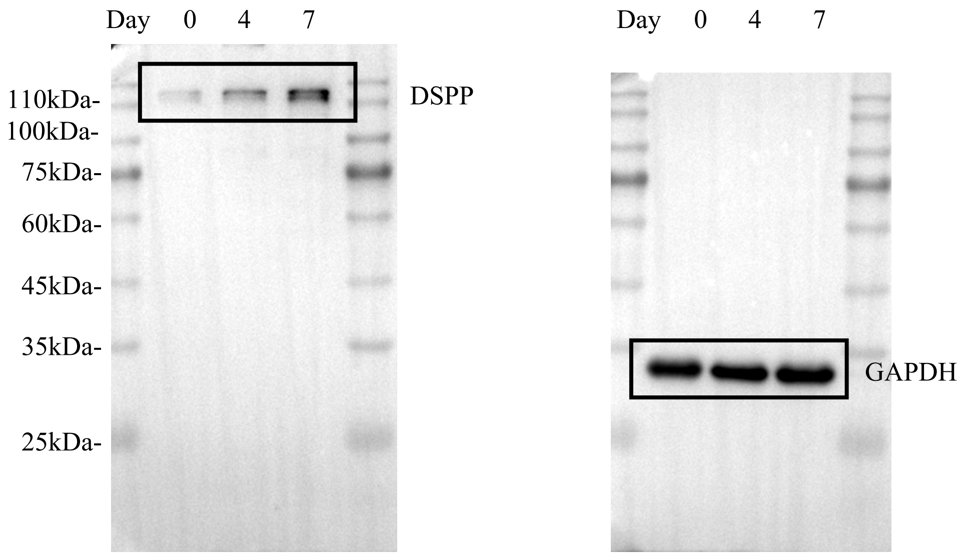

Supplementary Fig. 2B. Full-length blots/gels of SCUBE3 and DSPP elevated in hDPSCs after 7 d of being cultured in osteogenic inductive medium. Boxes areas are the samples we labelled.

Supplementary Fig. 3B

SCUBE3

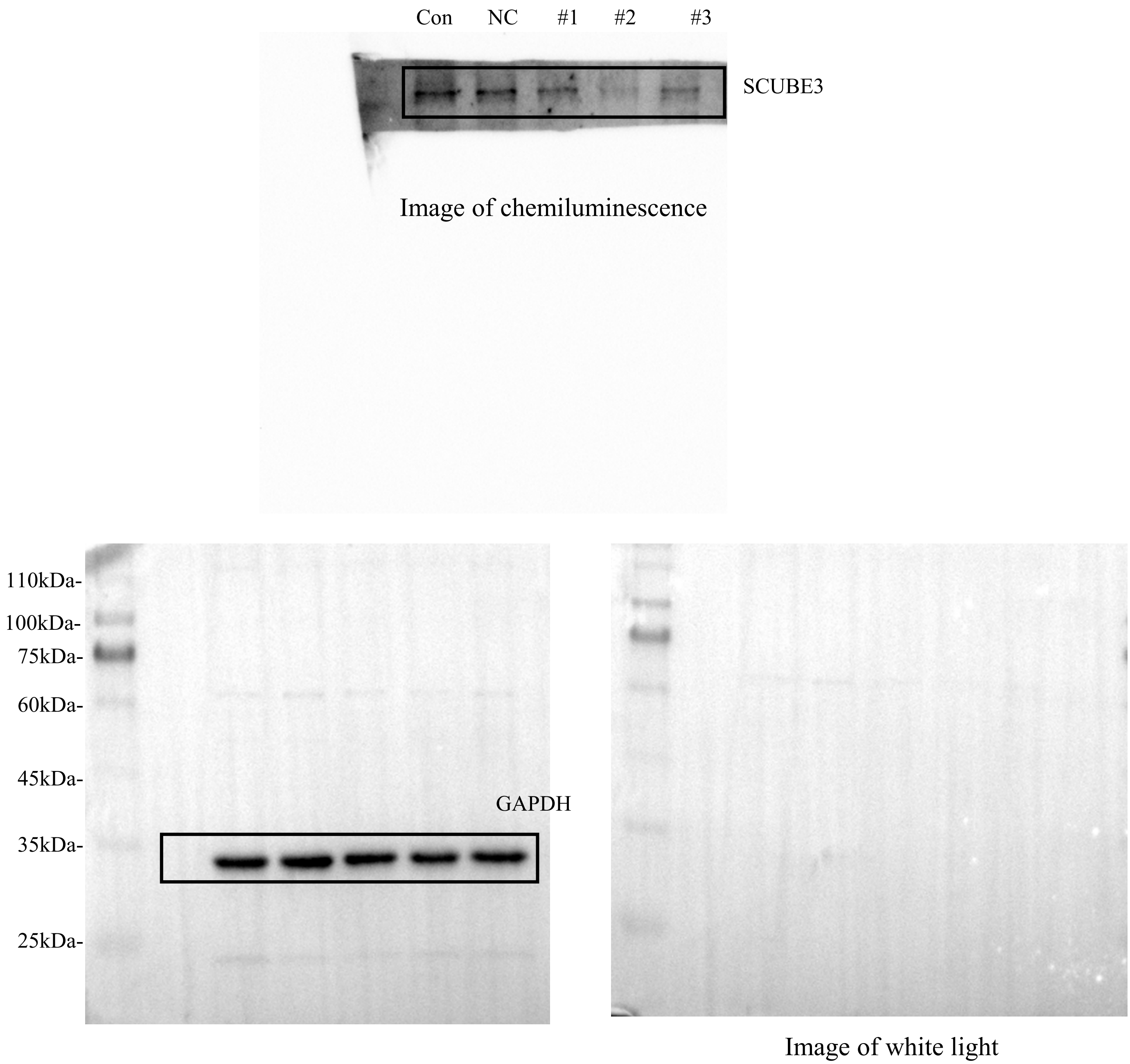

Supplementary Fig. 3B. Full-length blots/gels of SCUBE3 in hDPSCs transfected with either control or shRNAs. SCUBE3 is expressed in hDPSCs transfected with control shRNA, whereas its expression is drastically reduced in hDPSCs transfected with SCUBE3 shRNAs, with shSCUBE3#2 being the most effective shRNA. Boxes areas are the samples we labelled.

Supplementary Fig. 3D

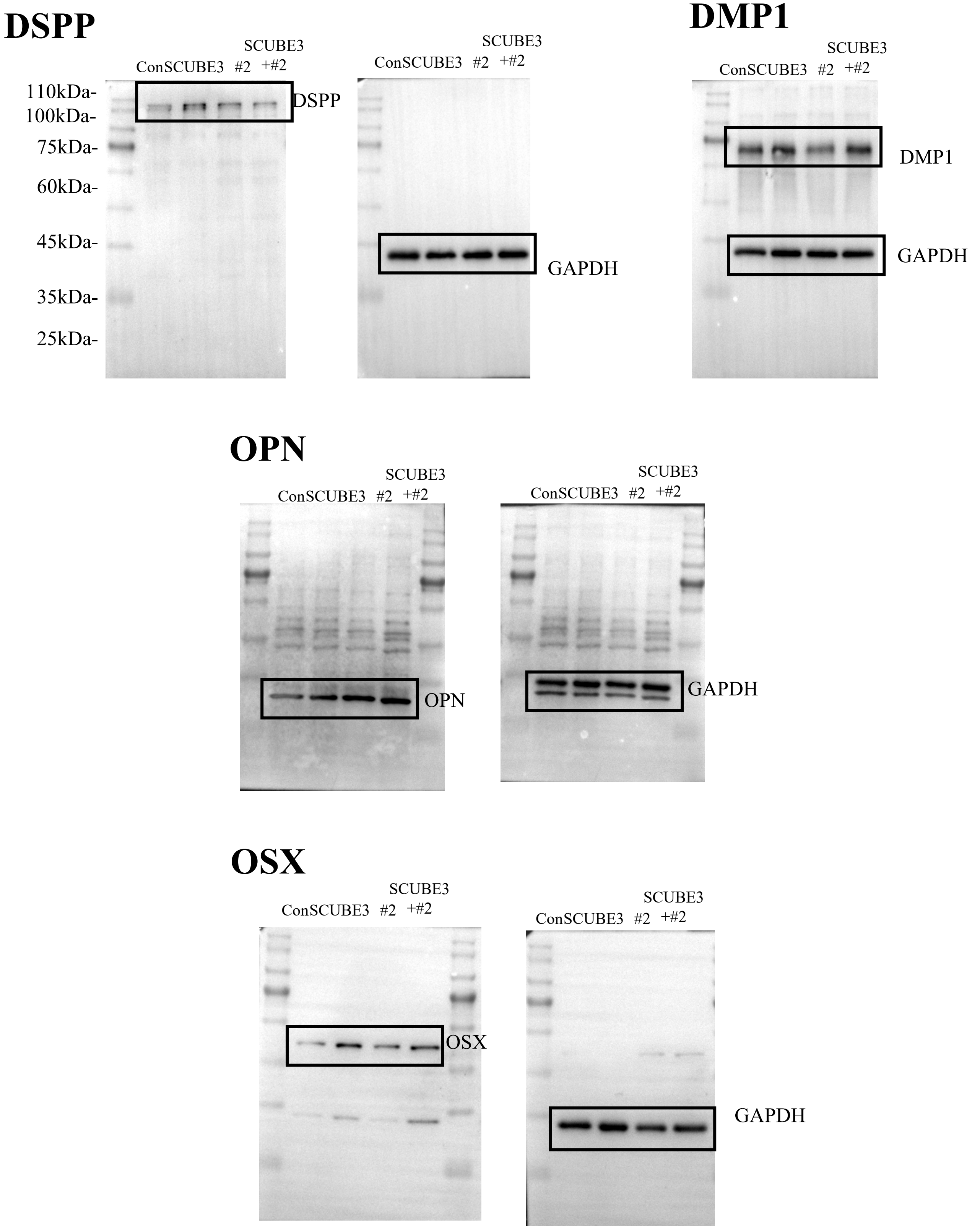

Supplementary Fig. 3D. hDPSCs were transfected with control shRNA or SCUBE3 shRNA#2 and with or without rhSCUBE3. Full-length blots/gels of the protein levels of odontoblastic differentiation markers in hDPSCs were evaluated by western blot. Boxes areas are the samples we labelled.

Supplementary Fig. 4B

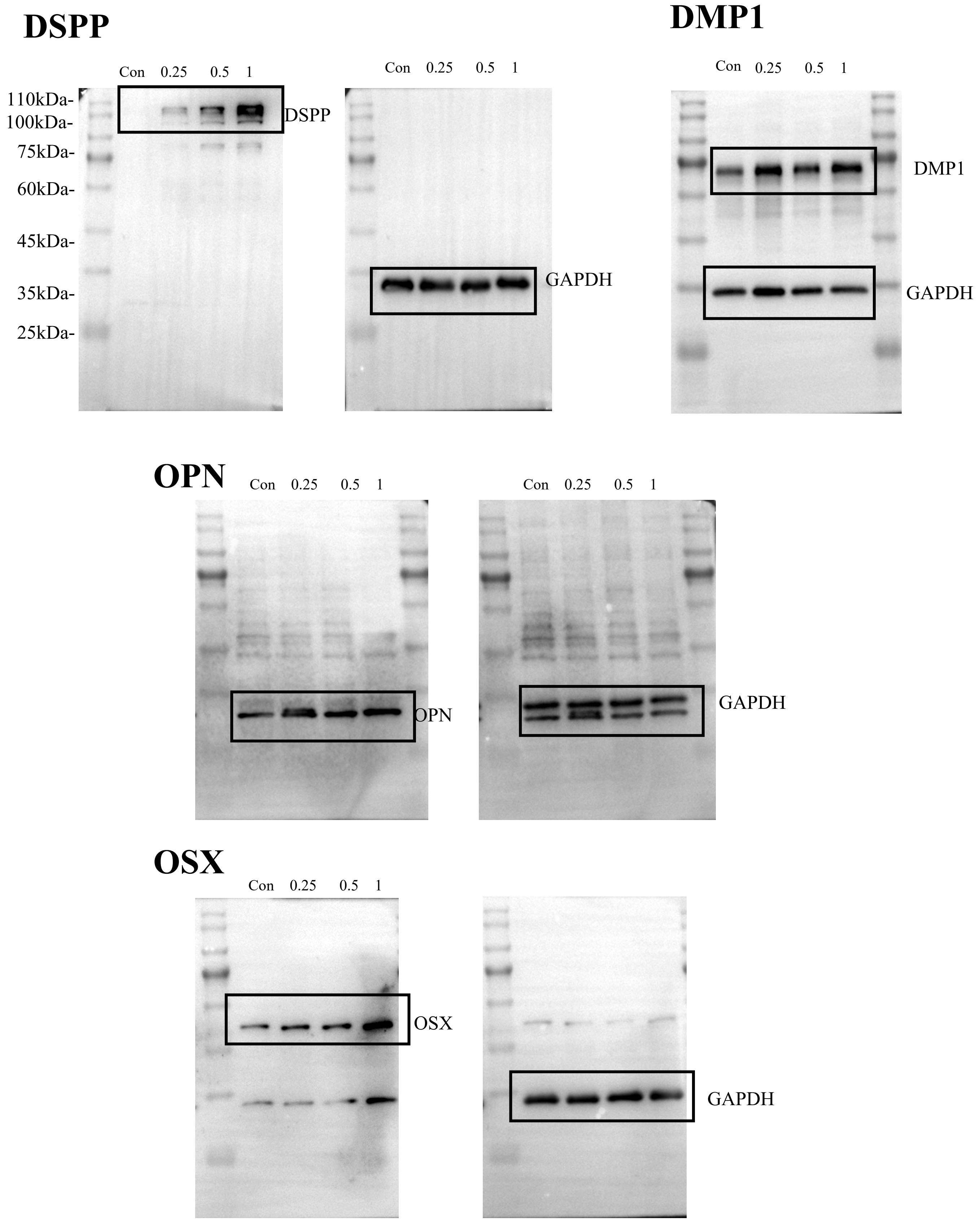

Supplementary Fig. 4B. hDPSCs were cultured in OIM add with 0, 0.25, 0.5, and 1 μg/ml rhSCUBE3 for 7 d. Full-length blots/gels of the expression levels of odontoblastic differentiation markers in hDPSCs are shown. Boxes areas are the samples we labelled.

Supplementary Fig. 6

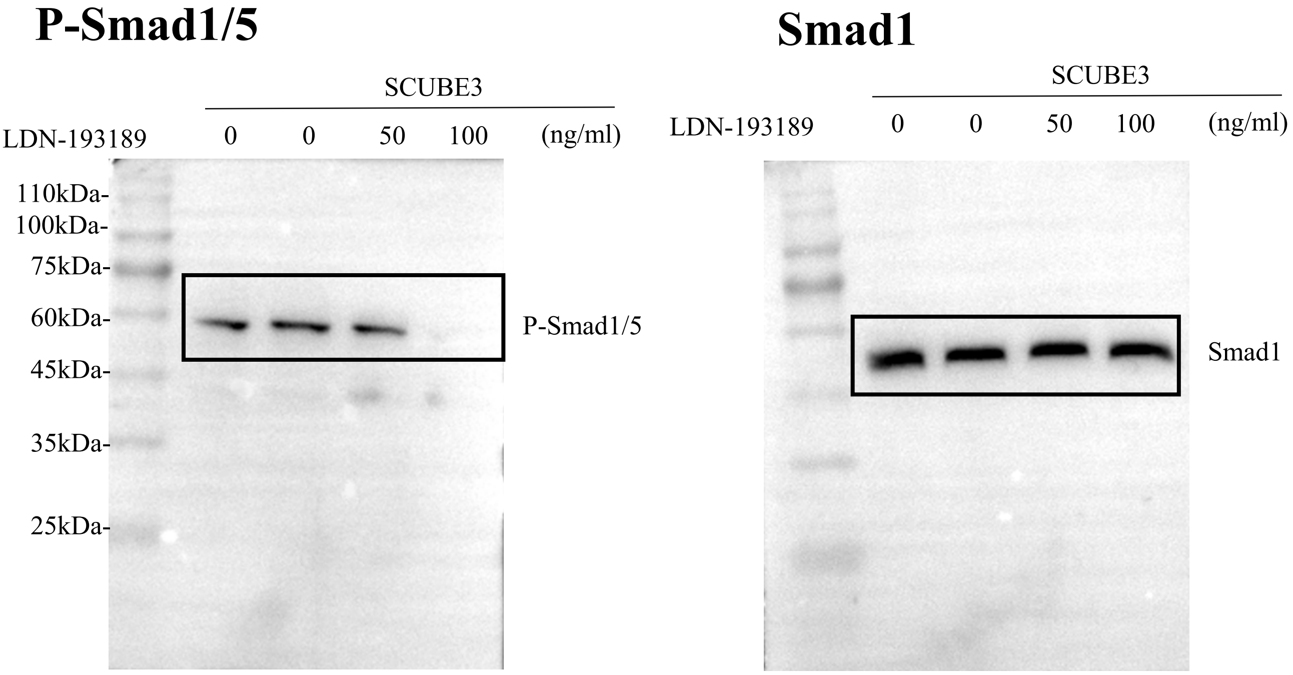

Supplementary Fig. 6. Western blotting was conducted to analyze the optimum concentration of LDN-193189, the BMP signaling pathway inhibitor at the protein level. Full-length blots/gels are shown. Boxes areas are the samples we labelled.

Supplementary Fig. 7A

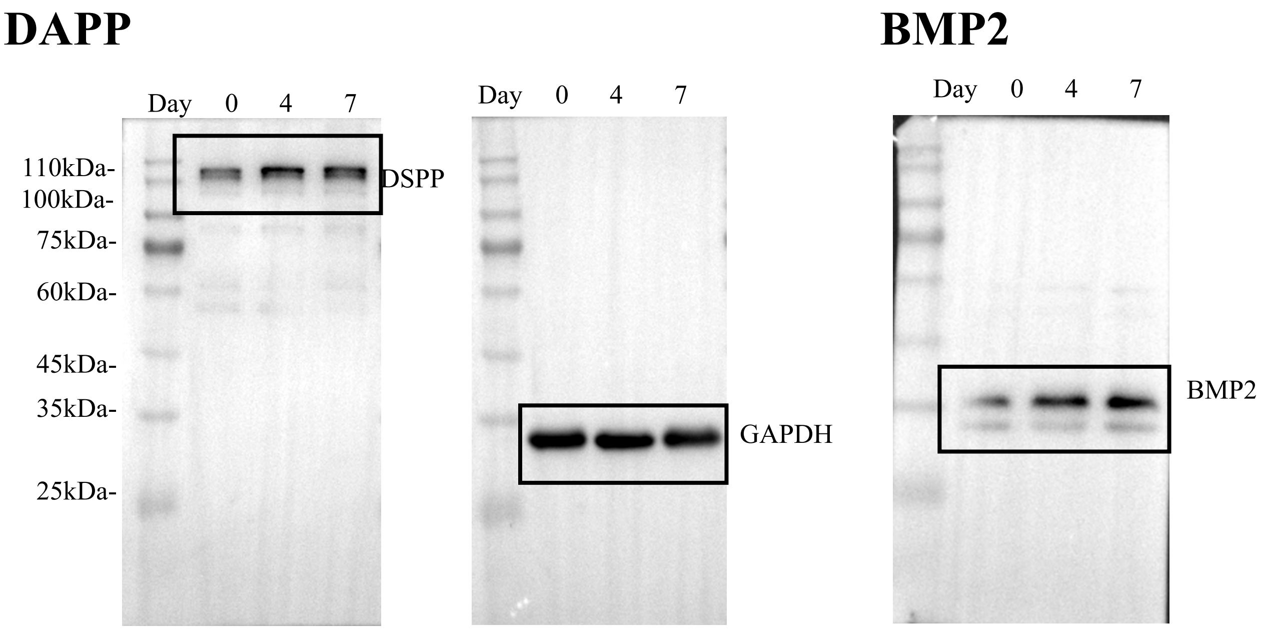

Supplementary Fig. 7A. BMP2 was increasingly expressed and released into the conditioned medium during odontoblastic differentiation of hDPSCs. Full-length blots/gels of DSPP and BMP2 levels in whole-cell lysates of hDPSCs on the indicated days during odontoblastic differentiation were shown. Boxes areas are the samples we labelled.

Supplementary Fig. 7B

SCUBE3

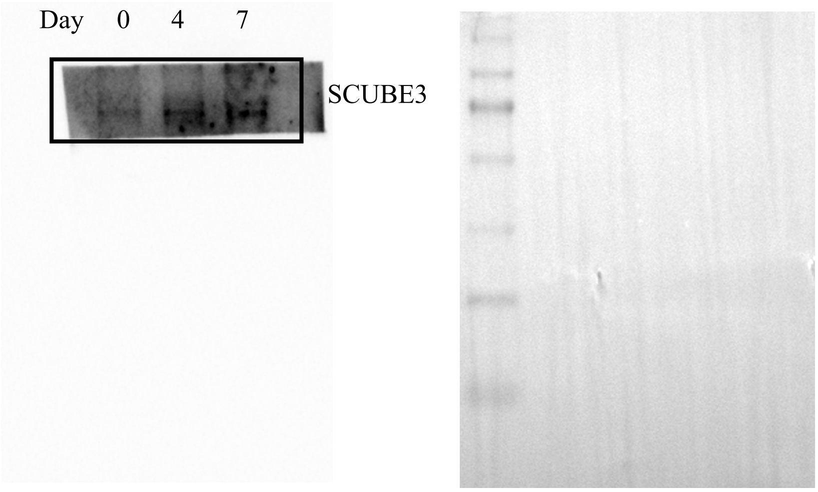

Image of chemiluminescence

Image of white light

BMP2

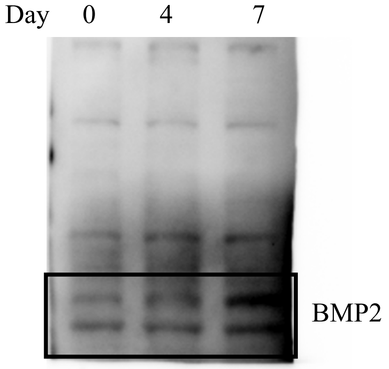

Image of chemiluminescence

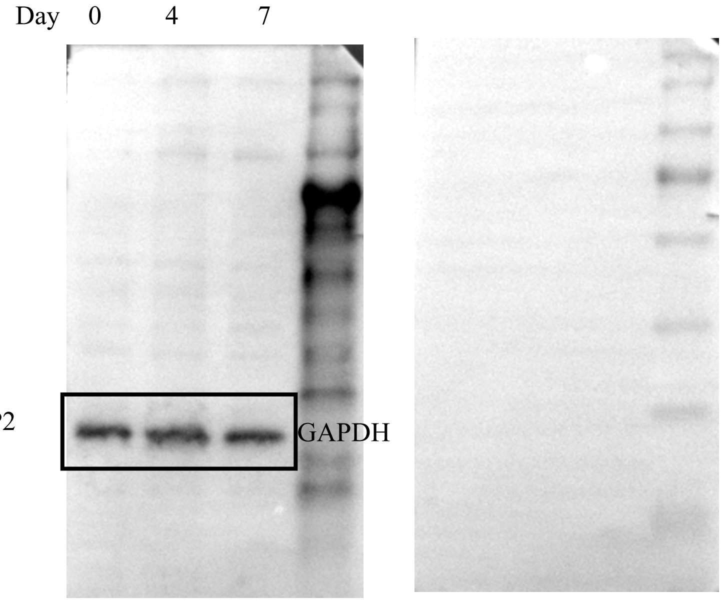

Image of white light

Supplementary Fig. 7B. Full-length blots/gels of secretory SCUBE3 and BM P2 protein in hDPSCs culture medium during odontoblastic differentiation. Boxes areas are the samples we labelled.
